# Local Drug Delivery Systems for Cancer Therapy

**DOI:** 10.1021/acsami.5c17220

**Published:** 2025-12-23

**Authors:** Zilong Jiang, Jiawei Zhang, Wei Cao, Yaqi Zhang, Yixian Cheng, Rui Fu, Weiwei Sheng, Bo Chen, Xianwen Wang, Guodong Cao

**Affiliations:** † Department of General Surgery, 36639 The First Affiliated Hospital of Anhui Medical University, Hefei, Anhui Province 230022, China; ‡ Department of Oncology, 36639 The First Affiliated Hospital of Anhui Medical University, Hefei, Anhui Province 230022, China; § Department of General Surgery, The First Affiliated Hospital of Nanjing Medical University, Nanjing, Jiangsu Province 210029, China; ⊥ The Endocrinology Department, 36639 The First Affiliated Hospital of Anhui Medical University, Hefei, Anhui Province 230022, China; ¶ School of Biomedical Engineering, Research and Engineering Center of Biomedical Materials, 12485 Anhui Medical University, Hefei 230032, China

**Keywords:** LDDS, hydrogel, scaffold, cancer therapy, PTT, CDT

## Abstract

Conventional methods of delivering anticancer drugs are typically not targeted and can lead to toxic side effects in normal tissues while maintaining effective drug concentrations in the tumor. Simultaneously, tumor barriers and rapid drug clearance pose challenges in maintaining effective drug concentrations in the tumor. Therefore, there is an urgent need for an oncology drug delivery method that offers precise targeting and minimal toxicity and side effects. Local drug delivery systems (LDDSs) are a class of fixed-domain tumor therapeutic systems with a broad range of applications. They exhibit robust drug-loading capabilities and can be implanted into tumor sites in a simple manner, such as through minimally invasive surgery. This enables them to release therapeutic agents in the tumor region in a sustained, controlled, and highly efficient manner. In addition, the characteristics of different types of LDDSs, such as deformability, adhesiveness, and responsiveness, enable them to provide a platform for synergistic tumor therapy, addressing various complex scenarios. In this review, we present commonly employed LDDSs and their therapeutic mechanisms, offering insights into potential directions for future development.

## Graphical abstract

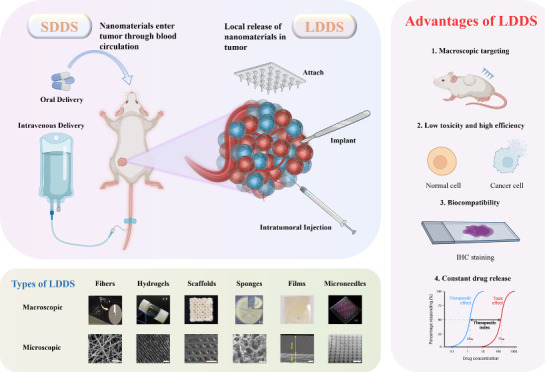

## 1 Introduction

Cancer is becoming the leading cause of death worldwide, with escalating morbidity and mortality rates over the decades. The efficient treatment of tumors has become an imperative challenge to address.^1–3^ At present, conventional drug delivery methods, such as intravenous drip and subcutaneous injection, are predominantly employed in tumor drug therapy. These methods facilitate drug distribution throughout the body via the blood circulation, offering benefits against tumor metastasis and recurrence.^4–6^ However, the nonspecific nature of these systemic drug delivery methods may lead to the local accumulation of the drug within the tumor and simultaneous drug dispersion throughout the body via the blood circulation. This may lead to toxic side effects and ultimately compromise the desired therapeutic outcome, representing a significant limitation in their utilization.^7,8^ Therefore, there is an urgent need for targeted drug delivery strategies that are minimally invasive, user-friendly, biocompatible, and capable of ensuring optimum therapeutic effects.

As a result, drug delivery systems (DDSs), defined by the National Institutes of Health as ″engineered technologies for targeted delivery and/or controlled release of therapeutic agents,″ have been proposed to improve drug delivery efficiency and ultimately enhance oncological outcomes.^9^ Compared with conventional drug delivery methods, DDS-based treatments can significantly diminish drug-related toxic side effects and yield superior therapeutic outcomes. Based on the mechanism of action and mode of administration, oncological treatments based on DDSs can be broadly classified as follows: systemic DDS (SDDS)-mediated systemic treatments and local drug delivery system (LDDS)-mediated local treatments.^10^

Briefly, SDDSs are drugs loaded or modified with nanomaterials, such as nanoparticles (NPs) or liposomes, to improve their pharmacokinetics, enabling delivery to tumor tissues throughout the body and facilitating therapeutic effects via the blood circulation after being injected intravenously or administered via alternative routes. LDDSs are macroscopic drug delivery platforms constructed using macromolecular polymers such as hydrogels and scaffolds. These systems possess the capacity to accommodate a substantial quantity of drugs. Upon implantation or injection into the tumor, they establish an in situ controlled-release drug storage and transport system, enabling sustained and controlled release of therapeutic agents. This approach not only locally targets the tumor but also promotes systemic tumor immunity by delivering immune factors or activating the immune system, leading to systemic tumor immune responses and the prevention of tumor recurrence and metastasis. Here, the advantages and disadvantages of conventional drug delivery methods and DDSs are initially contrasted. Subsequently, we provide a detailed review of the materials employed in common LDDSs, along with their therapeutic mechanisms, potential limitations, and future prospects.

## 2 Conventional Drug Delivery Methods vs DDSs

### 2.1 Deficiencies of Conventional Drug Delivery Methods

Taking chemotherapy as an example, traditional oncology chemotherapy is primarily administered intravenously to facilitate the distribution of chemotherapy drugs throughout the body via the blood circulation. This administration method plays a crucial role in eliminating cancer cells.^11,12^ However, owing to the poor tumor targeting of conventional chemotherapeutic agents, their intravenous administration leads to nonspecific distribution throughout the body. Consequently, this leads to systemic side effects and dose-limiting toxicity.^13,14^ Furthermore, most chemotherapeutic drugs that directly dissolve in the bloodstream are rapidly cleared. For example, nearly half of paclitaxel (PTX) is eliminated within the first 24 h after intravenous administration. This necessitates continuous intravenous infusion or multiple administrations to maintain effective drug concentrations, heightening the risk of toxic side effects and diminishing patient adherence and quality of life.^15^ Conversely, many chemotherapeutic drugs have markedly low solubility in water, hindering their suitability for intravenous delivery. Moreover, their modification to enhance solubility may reduce bioavailability, while dissolution in solutions containing surface-active substances can trigger sensitization reactions and other side effects.^16,17^ In addition, tumors possess natural barriers and an exceptional ability to adapt to their environment, enabling them to rapidly develop multidrug resistance (MDR) as a means of evading chemotherapeutic drugs. This further undermines the effectiveness of conventional chemotherapy treatments.^18^

### 2.2 Advantages and Disadvantages of SDDSs

Similar to traditional drug delivery methods, SDDSs primarily rely on intravenous administration. However, SDDSs are based on nano-DDSs, which have the capacity to enhance drug targeting. This enables preferential accumulation and release of the drug at the tumor site, consequently mitigating systemic toxicities, improving drug uptake efficiency, and safeguarding the active substances within from biological and chemical degradation.^19,20^ Commonly employed SDDSs, such as NPs, liposomes, and dendrimers, effectively target and deliver therapeutic drugs to tumor sites.^21,22^ Encapsulating drugs in nanocarriers offers many advantages, such as enhanced drug stability, prevention of blood degradation, targeted drug delivery, reduced toxic side effects, improved drug bioavailability, and even potential for overcoming MDR.^23–25^

Although SDDSs offer more precise targeting, fewer toxic side effects, and enhanced bioavailability than traditional drug delivery methods, the specific structure of tumors results in prolonged circulation of drug-loaded SDDSs in the bloodstream before they can localize in the tumor, leading to undesired inactivation of the drug and toxic side effects in normal tissues. Moreover, the use of biodegradable materials in SDDSs can lead to drug release in nontumor areas, which in turn can cause additional toxic side effects.^26^

### 2.3 Advantages of LDDSs Compare to SDDSs

Figure 1 illustrates the SDDS and LDDS modes briefly and summarizes the macroscopic and microscopic images of common LDDS.^27–35^ Unlike SDDSs, LDDSs require the implantation of carriers loaded with tumor therapeutic agents locally into the tumor. This enables continuous and efficient release of various therapeutic agents locally in the tumor, effectively eradicating the tumor while minimizing toxicity to normal tissues.^36^ In addition, LDDSs can be designed as controlled and/or sustained drug release systems that release therapeutic agents at predetermined rates for specific durations. This eliminates the need for repetitive drug administration, enhancing patient compliance.^37^ Sustained-controlled release formulations have been shown to increase local drug concentrations, thereby improving therapeutic efficacy. Consequently, LDDSs are considered a promising class of fixed-domain oncology therapeutic systems with a wide range of applications.

**Figure 1:**
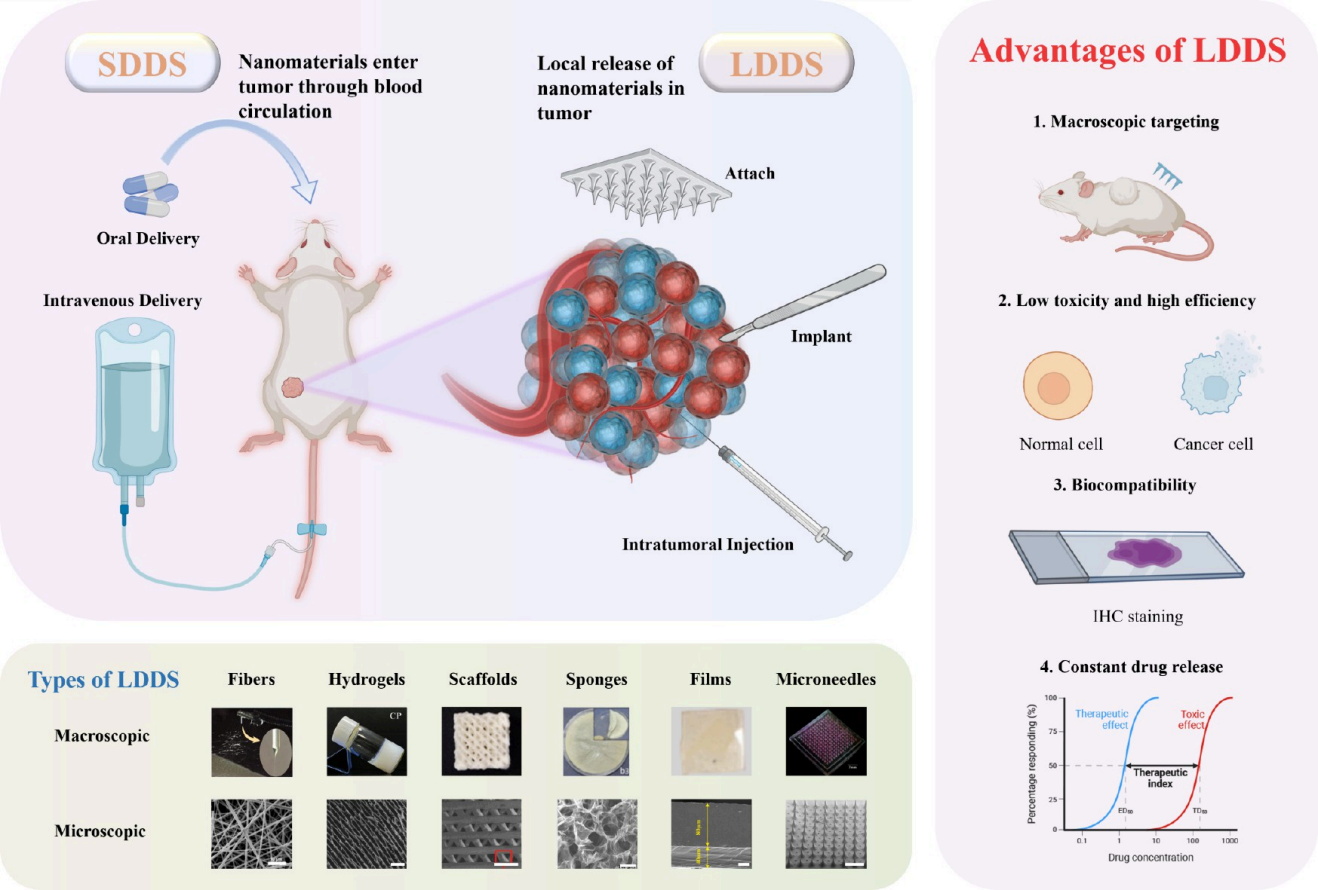
Schematic illustration of SDDS and LDDS (Partially created with bioRender.com). Images are reproduced with permission from the cited articles: macroscopic and microcosmic fibers (Scale bar: 10 μm),^43^ [Reproduced with permission from ref 43. Copyright 2024, Elsevier B.V.] macroscopic hydrogels,^33^ [Reproduced with permission from ref 33. Available under a CC BY license. Copyright 2021 Shaoyun Wang et al.] microcosmic hydrogels (Scale bar: 60 μm),^44^ [Reproduced with permission from ref 44. Copyright 2022, Wiley-VCH GmbH] macroscopic scaffolds,^29^ [Reproduced from ref 29. Copyright 2022 American Chemical Society] microcosmic scaffolds (Scale bar: 1 mm),^45^ [Reproduced with permission from ref 45. Copyright 2018, WILEY-VCH Verlag GmbH & Co. KGaA, Weinheim] macroscopic sponges,^34^ [Reproduced from ref 34. Copyright 2015 American Chemical Society] microcosmic sponges (Scale bar: 100 μm),^46^ [Reproduced with permission from ref 46. Copyright 2018, WILEY-VCH Verlag GmbH & Co. KGaA, Weinheim] macroscopic films,^27^ [Reproduced with permission from ref 27. Available under a CC BY license. Copyright 2020 Cárcamo-Martínez, A. et al.] microcosmic films (Scale bar: 20 μm),^47^ [Reproduced with permission from ref 47. Copyright 2025, Elsevier B.V.] macroscopic microneedles,^31^ [Reproduced with permission from ref 31. Copyright 2019, Elsevier Ltd.] and microcosmic microneedles (Scale bar: 1 mm).^48^ [Reproduced with permission from ref 48. Available under a CC BY license. Copyright 2025 Hang Yu et al.].

LDDSs offer several significant advantages compared with SDDSs:^3^ First, LDDSs enable macroscopic targeting of tumor therapeutics, allowing localized delivery of therapeutic agents to the tumor site. This enhances therapeutic efficacy and reduces the occurrence of toxic side effects. Second, LDDSs have the capability to form ″reservoirs″ of drugs locally, maintaining an effective drug concentration over an extended duration with a single application. Finally, the diverse variety and properties inherent to LDDSs allow them to address the varying requirements of tumor therapy, facilitating the selection of the optimal oncology treatment. Therefore, for local treatment of tumors, LDDS has significant advantages. More than that, LDDSs remain promising due to their diverse and exceptional properties. In the following sections, a few common LDDSs are introduced.

## 3 Types of LDDSs

LDDSs typically employ biodegradable materials as carriers for therapeutic agents and can be implanted into the tumor sites via injection or minimally invasive surgery. This approach enables localized, controlled therapy with minimal immune rejection or toxic side effects.^27,28^ Materials utilized in LDDSs must exhibit several characteristics:^3,10,38,39^ 1) Superior biocompatibility to minimize the body’s immune rejection. 2) Biodegradability, thus obviating the need of surgical removal 3) Capacity to accommodate substantial quantities of tumor therapeutic agents. 4) Ability to achieve constant or controlled drug release. 5) Accessibility and cost-effectiveness. These characteristics have spurred the development of numerous excellent materials for LDDSs, with fibers, hydrogels, scaffolds, etc. gaining substantial research attention.^3,36,40^ These materials are not only employed for local delivery of cancer therapeutic agents but also exhibit remarkable potentiality in innovative cancer treatment approaches, such as domain-specific photothermal therapy and immunotherapy. Therefore, LDDSs are considered a class of oncology therapeutic systems with promising applications.^41,42^ Following, we will summarize the main forms of LDDS in the current research.

### 3.1 Fibers

Fibers encompass a wide range of natural or synthetic filamentous solids, each possessing different properties. Most fibers utilized in LDDSs share common features, such as a large specific surface area and high porosity.^49^ In addition to readily available natural fibers, such as silk and spider silk, LDDS fibers are mostly synthesized through electrostatic spinning. This process entails preparing an electrostatic spinning precursor solution, followed by electrostatic spinning using an electrostatic spinning device under specific parameters. The resultant fibers require additional processing, such as static or drying treatments.^50–55^

Silk, being a common natural fiber, possesses characteristics such as ready availability, ease of sterilization, superior biocompatibility, and the capacity for straightforward modification and processing to create excellent LDDSs.^36,56^ Electrospun fibers, characterized by their substantial specific surface area and high porosity, can be directly used as carriers for various drugs and molded into specific shapes. In addition, their release behavior can be controlled by modifying the preparation method.^57–60^ Moreover, compared with natural fibers, electrospun filaments exhibit customizable material properties. Various therapeutic agents, ranging from small-molecule drugs to biomacromolecules, such as antibiotics, proteins, DNA, small interfering RNA (siRNA), and oligo/peptides, have been successfully incorporated into electrospun fibers.^61^ For example, Kuang et al.^62^ employed hydrophilic poly­(ethylene oxide) (PEO) and hydrophobic poly­(l-lactide) (PLA) to create doxorubicin-loaded (DOX-loaded) PEO10-PLA90 cospun electrospun fibers with typical two-phase release kinetics. These fibers have been utilized as two-phase DDSs for localized oncology therapy. Amna et al.^63^ pioneered the fabrication of nanofiber mats composed of poly­(epsilon-caprolactone) (PCL) mixed with camptothecin through a one-step sol–gel electrospinning process. In vitro release experiments showed that drug release from such fiber mats occurs in two phases: an initial burst release (approximately 17% of total drug release), followed by a sustained, gradual release of the drug, thus enhancing drug utilization.

In general, fibers serve as implantable and user-friendly LDDSs with superior biocompatibility, biodegradability, mechanical properties, and robust plasticity. This makes them highly promising for tumor treatment and prevention.^64–67^ However, the linear structure of drug-loaded fibers complicates controlled drug release. Consequently, these fibers are not employed individually but rather woven into two-dimensional fiber mats or incorporated into three-dimensional (3D) scaffolds, sponges, etc.

### 3.2 Hydrogels

Hydrogels are generic terms for 3D networks of polymers cross-linked to maintain their structure in water or biological fluids, absorbing substantial liquid quantities without dissolution.^68^ Hydrogels consist of polymeric materials in the form of solutions, suspensions, or semisolids.^69^ They undergo an in situ phase change following administration, transforming from a solution or suspension into a semisolid or solid state; thus, hydrogels combine the advantages of both gels and solutions.^70,71^ Consequently, hydrogel-based LDDSs exhibit characteristics such as high local drug concentration, minimal in vivo dosage, extended drug retention times, ease of combining multiple therapies, and favorable patient compliance. These qualities have made hydrogel-based LDDSs a prominent research focus in the treatment of tumors.^72–74^

Although hydrogels vary in type, raw materials, and synthesis methods, their general synthesis involves combining a substrate and a cross-linking agent. For instance, consider alginate (ALG) hydrogel synthesis. The steps involve roughly dissolving alginate in an appropriate amount of water to form a sol, followed by the introduction of a sufficient cross-linking agent, such as Ca^2+^, to induce a phase change from sol to a solid gel state (Figure 2 A). Basic hydrogels can be injected into the body in the sol state and then form a gel in situ. This approach offers the advantages of simplicity and ease of synthesis but lacks adaptability to the environment, which can limit its application. Therefore, at present, responsive hydrogels are commonly utilized, which are primarily of two types: pH-sensitive and temperature-sensitive.

**Figure 2:**
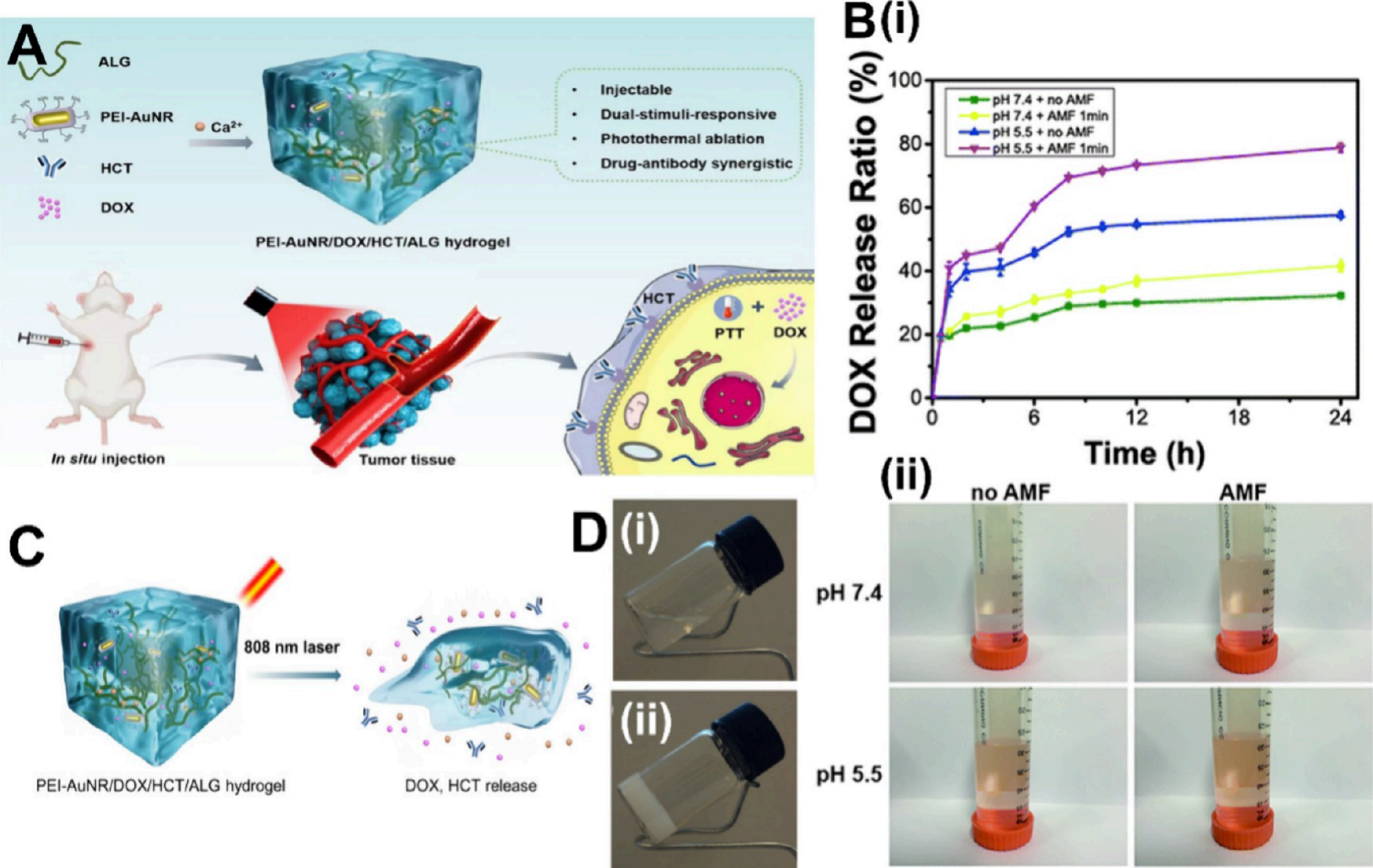
Common responsive hydrogels. (A) In situ forming PEI-AuNR/DOX/HCT/ALG hybrid hydrogel for photothermal ablation and antibody-drug synergistic therapy of HER-2 overexpressing breast tumor.^75^ Abbreviations: AuNR, gold nanorod; HCT, Herceptin; HER-2, human epidermal growth receptor 2; PEI, polyethylenimine. [Reproduced with permission from ref 75. Available under a CC BY license. Copyright 2022 Li Zhao et al.] (B) (i) In vitro release profiles of pH-sensitive hydrogels at different pH values (pH 7.4 and pH 5.5) with or without the alternating magnetic field (AMF), showing that DOX was released at pH 5.5 after exposure to the AMF for 1 min. (ii) The corresponding digital photos of the cumulative release of DOX in vitro at 24 h.^76^ [Reproduced with permission from ref 76. Available under a CC BY-NC 3.0 license. Copyright 2018 X. Zhou et al.] (C) Schematic diagram of DOX and HCT release from PEI-AuNR/DOX/HCT/ALG hydrogel under 808 nm laser irradiation. (D) Photographs of the sol–gel transition for temperature-sensitive hydrogels (i) at 25 °C and (ii) at 40 °C.^77^ [Reproduced from ref 77. Copyright 2019 American Chemical Society].

pH-sensitive hydrogels typically contain either −COOH or -NH_3_ groups, forming ions that are distinct from the surrounding pH level, thereby facilitating the contraction or swelling of the hydrogel. For example, in alkaline environments, −COOH groups ionize into −COO^–^ groups, and the electrostatic repulsion between these −COO^–^ groups causes the polymer chains in the hydrogel to stretch. Consequently, the hydrogel swells as water enters its 3D lattice, facilitating the release of loaded drugs. In acidic conditions, the −COOH groups may form new hydrogen bonds, and the complex hydrogen bonding promotes the shrinkage of the hydrogel and hinders water absorption, thereby making it challenging to release the loaded drug. Hydrogels enriched with -NH_3_ groups exhibit the opposite behavior to those enriched with −COOH groups, shrinking in alkaline environments and swelling in acidic conditions.^78^ Figure 2 B illustrates a pH-sensitive hydrogel that promotes the release of agents in acidic environments. Xie et al.^79^ synthesized various innovative pH-sensitive polymers for controlled drug release through free radical polymerization, including Chinese quince seed gum and poly­(N,N-diethylacrylamide-*co*-methacrylic acid). They experimentally demonstrated that all gel polymers exhibit a significant increase in swelling as the pH level increases from 1.2 to 7.4 at 37 °C, suggesting that these hydrogels have potential applications in transoral intestinal drug delivery.

Temperature-sensitive hydrogels can be categorized into two distinct types. In one type, the chemical bonds (primarily hydrogen bonds) between the polymers that form the hydrogel are disrupted due to increased temperature, leading to a loss of the primary support between the polymers. Consequently, this results in a reduction in the volume of the hydrogel and the expulsion of water, subsequently facilitating the release of the loaded drug (Figure 2 C).^80–82^ The other type of temperature-sensitive hydrogel remains in a liquid or semisolid state at low temperatures. Upon administration of the hydrogel or drug loaded hydrogel into the body, as the temperature gradually increases from room temperature to body temperature, the hydrogel undergoes a phase transition at the application site, transforming from a liquid or semisolid state into a hydrogel (Figure 2 D). This transition promotes robust adhesion and delayed drug release.^83,84^ For example, Lin et al.^85^ developed a temperature-sensitive hydrogel system using thermosensitive Pluronic F127, which remains a clear liquid at 4 °C but transitions into a gel state in less than a minute when warmed to 37 °C. Furthermore, experiments have demonstrated that intratumoral administration of this hydrogel results in linear drug release and in vivo elimination, with a slower drug delivery rate, a higher drug retention rate, and the formation of a slow-release reservoir in vivo, ultimately showcasing its favorable anticancer effects.

Hydrogel DDSs offer a convenient means to deliver drugs to the desired site in the patient, establishing drug reservoirs to reduce the frequency of drug administration throughout the treatment process.^86–91^ Simultaneously, hydrogels enable sustained release of therapeutic agents in target tissues, thereby mitigating systemic adverse effects and off-target toxicity, underscoring their excellent suitability for oncology therapy.^92,93^ However, the mechanical strength of most hydrogels is poor. In cases where hydrogels undergo compression after curing in vivo due to patients’ daily activities, there is a risk of excessive release of loaded therapeutic agents or even rupture of the hydrogel. This could lead to adverse reactions and compromise subsequent oncological treatments.

### 3.3 Scaffolds

Scaffolds for LDDSs are constructed using degradable polymers, primarily extracellular matrix components such as alginate, collagen, and hyaluronic acid, along with chitosan and synthetic materials such as poly­(lactic acid) and poly­(vinyl alcohol).^94,95^ These materials are interwoven with each other, forming a 3D lattice structure that serves as the scaffold’s core. This structure not only provides support but also functions as a carrier for drugs and facilitates cell growth.^96^ Combining certain materials with specific NPs or natural polymers can further improve the properties. Composite scaffolds offer advantages including high purity, high porosity, a large specific surface area, nontoxicity, and superior biocompatibility.^97^

To achieve a homogeneous scaffold structure, scaffolds are typically synthesized through either 3D printing technology or electrostatic spinning technology, both of which generally involve first synthesizing the precursor solution for the scaffolds, followed by scaffold preparation using 3D printing equipment or electrostatic spinning equipment.^98^ Subsequently, processes such as freeze-drying, vacuum-drying are performed. Drugs can be incorporated either in the precursor solution or post-scaffold fabrication, utilizing methods such as drug immersion.^99–101^ Lin et al.^102^ used chitosan-coated poly­(lactic acid) nanofibers with mineralized hydroxyapatite as a scaffold material for bone tissue engineering. This chitosan scaffold, featuring hydroxyapatite, exhibits suitable roughness, superior biocompatibility, and a bone-like structure, rendering it a promising biomaterial for bone regeneration and the enhancement of bone mineralization. Thakur et al.^103^ dissolved lidocaine and mupirocin in polylactic acid solutions, respectively, and subsequently constructed a scaffold system using a dual-nozzle electrostatic spinning technique. In vitro drug release experiments revealed a staged drug release pattern attributable to the distinct release characteristics of the two drugs. Meng et al.^101^ explored the effect of material and nanofiber arrangement on scaffolds. They prepared poly­(d,l-lactide-*co*-glycolide) (PLGA)/chitosan nanofibrous scaffolds through directional electrospinning and random electrospinning, respectively. Subsequently, fenbufen was employed as the loaded drug, and its release behavior porosity was explored by altering the chitosan ratio and electrospinning technique. The results showed that an increase in chitosan content leads to an enhanced drug release rate, attributed to the improved hydrophilicity of PLGA/chitosan composite scaffolds due to chitosan incorporation. Moreover, PLGA/chitosan nanofiber scaffolds produced by directional electrospinning exhibit a lower drug release rate compared to randomly oriented PLGA/chitosan nanofiber scaffolds. This indicates that the ratio of materials and the arrangement of nanofibers affect the drug release kinetics of these scaffolds, providing researchers with a method for controlling drug release rates in drug-loaded scaffolds.

Scaffolds possess excellent features, such as efficient drug loading capacity, controlled drug release profiles, and the capacity to emulate natural tissues to enhance immune responses.^104^ Compared with systemic therapy, biomaterial-based scaffolds require lower drug dosages and offer sustained and controlled drug release, demonstrating significant potential in biomedical applications.^105^ Furthermore, certain scaffolds exhibit physicochemical and biological properties that not only modify the immune microenvironment and inhibit tumor formation but also provide a suitable environment for immune cell interaction, expansion, and proliferation in both in vitro and in vivo settings.^106–109^ These properties render them excellent carriers of immunomodulatory vaccines, with the release of immunotherapeutic agents typically occurring through diffusion and scaffold degradation. This is exemplified by PLGA scaffolds, which have been extensively investigated as vaccine carriers.^110–112^ Despite the potential of scaffolds to influence immunity, their application require additional invasive procedures. Consequently, scaffolds are currently commonly employed for the postoperative removal of residual tumor cells and the prevention of tumor recurrence.^106,113^

### 3.4 Sponges

Sponges are typically composed of cross-linked macromolecules and are highly porous and elastic solids, featuring numerous vesicular microporous structures and small pore openings on the surface that facilitate the absorption of substantial liquid volumes. Sponges commonly used in LDDSs can be categorized as natural polymer sponges or artificial polymer sponges. Most of them adopt a sandwich-like structure, typically with precured hydrogel-like material as the bottom and top layers of the sponge, with an electrospun fiber mat serving as the interlayer. After complete gel curing, these sandwich-like structures are subjected to freeze-drying to obtain the final product (Figure 3 A).^114–117^

**Figure 3:**
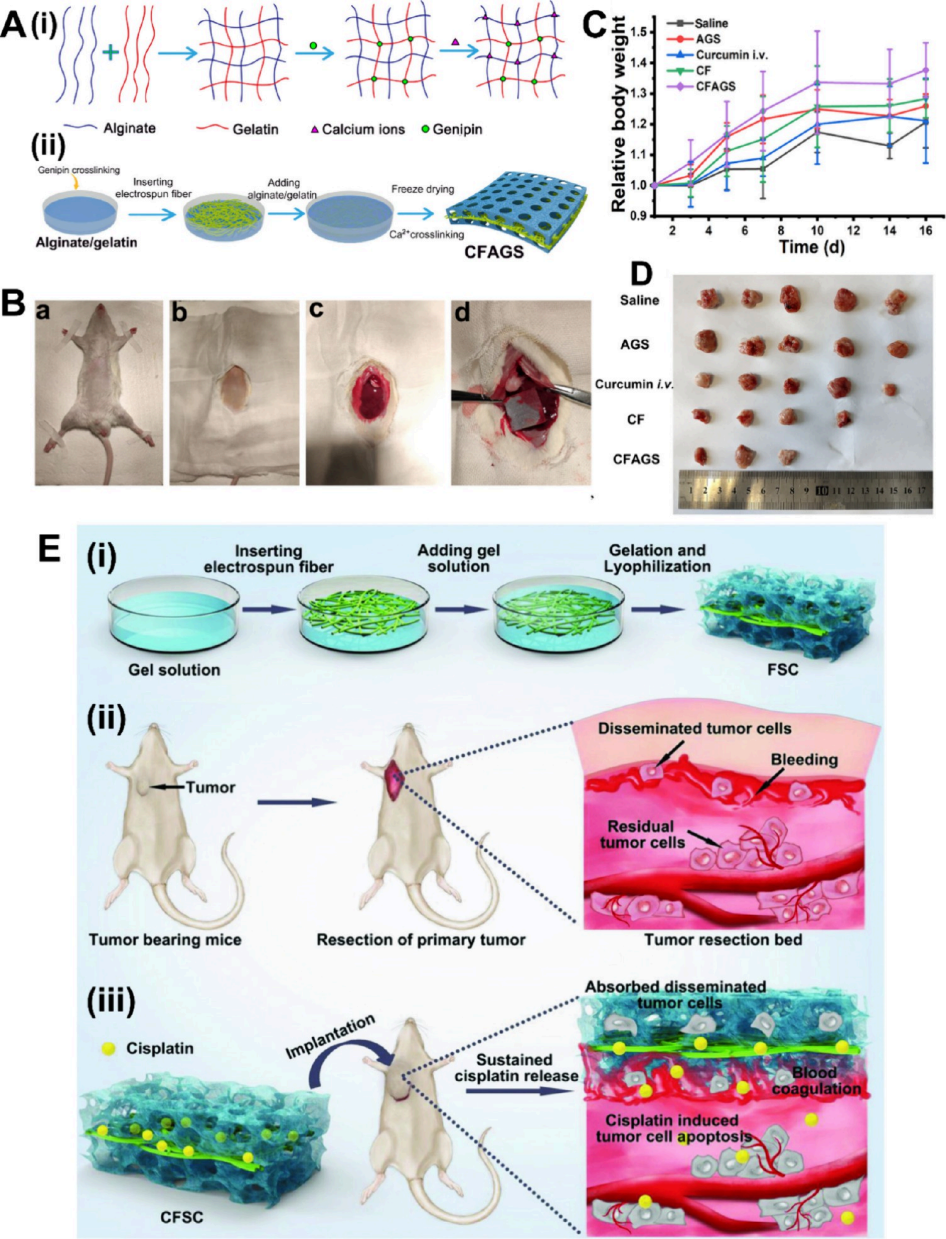
Preparation and applications of common sponges. (A) (i) Schematic illustration of interpenetrating polymer network (IPN) alginate/gelatin sponges (AGS) using CaCl_2_ and genipin as cross-linking agents. (ii) Schematic illustration of a novel alginate/gelatin sponge combined with curcumin-loaded electrospun fibers (CF). (B) Hemostatic experiments in vivo: Photographs of AGS in the liver of rat hemostasis models. (C) The relative body weights variations in mice after tumor removal surgery. (D) Excised recurrent solid tumors from different treatment groups on the 16th day.^117^ [Reproduced with permission from ref 117. Copyright 2021, Elsevier B.V.] E) (i) Illustration of preparation procedure of FSC. (ii) Illustration of establishment of subcutaneous postoperative tumor model and microenvironment of tumor resection bed. (iii) CFSC promotes blood coagulation and induces apoptosis of disseminated and residual tumor cells. [Reproduced with permission from ref 46. Copyright 2018, WILEY-VCH Verlag GmbH & Co. KGaA, Weinheim].

Alginate sponges exhibit notable gelation properties, with the calcium ions they contain capable of exchanging with sodium ions in the blood, resulting in the formation of a sodium alginate gel that covers the wound surface. The exchanged calcium ions enter the bloodstream to activate coagulation factors in the blood, thus achieving rapid hemostasis.^118,119^ Chen et al.^117^ developed a novel alginate/gelatin sponge compounded with curcumin electrospun fibers using electrostatic spinning and interpenetrating polymer network strategies involving CaCl_2_ and genipin cross-linking, respectively. Subsequent experiments showed that this preparation approach improved the sponge’s water resistance and hemostatic efficacy (Figure 3 B), surpassing commercially available gelatin hemostatic sponges.^120–122^ In a subcutaneous postoperative recurrence model, this sponge exhibited controlled curcumin release, leading to curcumin accumulation at the tumor surgical site, thereby inhibiting local tumor recurrence (Figure 3 C, 3 D). Zhang et al.^46^ also developed a sandwich-like cisplatin-loaded fiber/sponge composite (CFSC) with dual functions of chemotherapy and hemostasis (Figure 3 E). The gelatin/chitosan-based sponge layer on its surface significantly reduced intraoperative bleeding and absorbed disseminated tumor cells. The cisplatin within the interlayer fibers eliminated the absorbed tumor cells within the sponge as well as residual tumor cells postsurgery, thereby reducing the risk of recurrence and metastasis. Additionally, they demonstrated that CFSC significantly inhibited both local recurrence and distant metastasis of tumors in a subcutaneous tumor recurrence and metastasis model. Liu et al.^123^ developed a gelatin sponge delivery system incorporating polylactide-*co*-glycolide PTX microspheres (PLGA–PTX) as a treatment for lymphatic metastasis in lung cancer. This system exhibited slow-release properties in vitro and targeted the lymphatic system in a rat model. The PLGA–PTX-loaded lymphatic drug exposure increased 100- to 400-fold compared to intravenous injection. Moreover, an in vivo model demonstrated that the system was capable of significantly lowering peak blood concentrations, minimizing systemic toxicities, and markedly decreasing lymphatic metastasis in lung cancer.

Evidence suggests that bleeding during tumor resection can lead to the dissemination of tumor cells into the bloodstream, elevating the levels of circulating tumor cells (CTC) and consequently increasing the risk of both local recurrence and distant metastasis.^124,125^ Although the robust ability of sponges to absorb and stop bleeding can markedly reduce the risk of tumor recurrence and metastasis due to hemorrhage, they exhibit poor mechanical properties and susceptibility to deformation and rupture when subjected to pressure, limiting their utility in hemostasis of large wounds. In addition, sponges can absorb substantial quantities of oncology chemotherapeutic agents, rendering them suitable carriers for various chemotherapeutic agents and thereby reducing the number of drug carriers. Simple compounding and modification of sponges can also yield favorable antimicrobial activity, adsorption capabilities, and tumor cell eliminating effects, ultimately achieving the goal of preventing tumor recurrence and metastasis.^116,126^ Therefore, sponges hold substantial potential in tumor treatment and postoperative care.^127^

### 3.5 Films

LDDS films consist of biocompatible and biodegradable materials with diverse properties, such as ductility, viscosity, and plasticity, depending on the selected material. Most films are prepared through casting synthesis, i.e., casting a prepared precursor solution onto a flat surface to produce a homogeneous liquid film, which is then dried, either directly or after solidification.^27,56,128,129^

Delivering drugs to the soft tissue surgical margins of tumors presents a significant challenge due to their heightened risk of local tumor recurrence. Moreover, most soft tissue margins are irregular, which limits the extensive utilization of rigid polymers. Achieving adequate surface coverage, immobilization, and drug diffusion in such cases proves to be a daunting task for other DDSs. While sprays, adhesives, or hydrogels may attain acceptable drug coverage, they fall short in terms of controlled drug release and mechanical stability. In contrast, films can be sutured to any desired site, addressing the issues of dislodgment commonly encountered with other DDSs in such scenarios. This approach demonstrates promise for achieving localized and sustained delivery of cancer chemotherapeutic drugs, establishing itself as an emerging and innovative DDS.^38,130^

Costa et al.^131^ demonstrated that the limited surface area and permeability of the oral mucosa, along with continuous saliva secretion, limit the efficacy of oral ointments and gels for drug delivery. As a response to this limitation, they designed a chitosan film loaded with 5-aminolevulinic acid for photodynamic therapy (PDT) of oral cancer, which exhibited robust adherence to the oral mucosa and ensured stable drug delivery. Amatya^132^ prepared a gelatin film loaded with bovine serum albumin (BSA)/silver NPs (AgNPs) with excellent solubility, mechanical properties, bioadhesion, and compatibility. The BSA/AgNPs composite exhibited superior photothermal activity and demonstrated remarkable in vivo anticancer effects, significantly inhibiting tumor growth with a single administration. Kim et al.^133^ engineered a chitosan film loaded with ellagic acid and employed WM115 melanoma in in vivo experiments. The results revealed the film’s potent inhibitory effect on melanoma growth, ultimately inducing cell death. Dhanikula et al.^134^ prepared chitosan films loaded with PTX using the casting method. PTX demonstrated potent tumor-eradicating capabilities through an initial burst release followed by sustained release as the film degraded in vivo. Liu et al.^135^ prepared poly­(glycerol monostearate co-e-caprolactone) polymer films loaded with PTX as a chemotherapeutic agent. They established a Lewis lung carcinoma-resected mouse model and performed postoperative implantation of these PTX-containing films. A control experiment was conducted using a group of mice implanted with PTX-free films and another group receiving PTX via intraperitoneal or subcutaneous injection. The results showed that none of the 11 mice receiving PTX-containing films experienced tumor recurrence in the film-covered area. Furthermore, more than 80% of these mice survived for more than 90 days. In contrast, the control mice exhibited substantially high rates of tumor recurrence and mortality, with all control mice experiencing a mortality rate exceeding 70% within 20 days. These results underscore the biocompatibility of the film and its effectiveness in preventing tumor recurrence.

Compared with other LDDSs, films offer distinct advantages. They can perfectly fit surgical incisions, eliminating concerns about detachment. Additionally, film modification can promote wound healing and prevent infections, and the films’ high drug loading capacity with controlled release can markedly improve drug utilization while minimizing systemic adverse effects.^136–138^ However, the application of thin films is limited. They are mostly used for the treatment of internal margins after surgical tumor resection and the prevention of tumor metastasis or recurrence. Hence, the development of thin films is limited to a certain extent.

### 3.6 Microneedle-Based Systems

Microneedle-based systems, primarily microneedle patches, comprise two components: the microneedle array and the substrate.^139^ The microneedle array is composed of an arrangement of cones or triangular cones of the same size that are evenly spaced and exhibit a certain degree of mechanical strength. In contrast, the substrate typically consists of a pliable and moldable film patch.^140–142^ Microneedle arrays, exemplified by microneedle patches, are predominantly prepared using the casting method. This method involves loading the microneedle precursor solution into the desired microneedle molds, pouring it into the prepared substrate solution, curing, removing, and further processing as required to yield microneedle patches (Figure 4 A).^143–146^ For addressing superficial tumors, such as skin cancer, the epidermis represents a key impediment to therapeutic interventions.^147^ Microneedle-based systems serve as drug delivery platforms, featuring a substrate and its supporting microneedle array with sufficient mechanical strength to pierce the skin barrier.^148^ These systems effectively deliver small-molecule therapeutic agents subcutaneously, rendering them a subject of extensive research in the context of transdermal drug delivery.^149^

**Figure 4:**
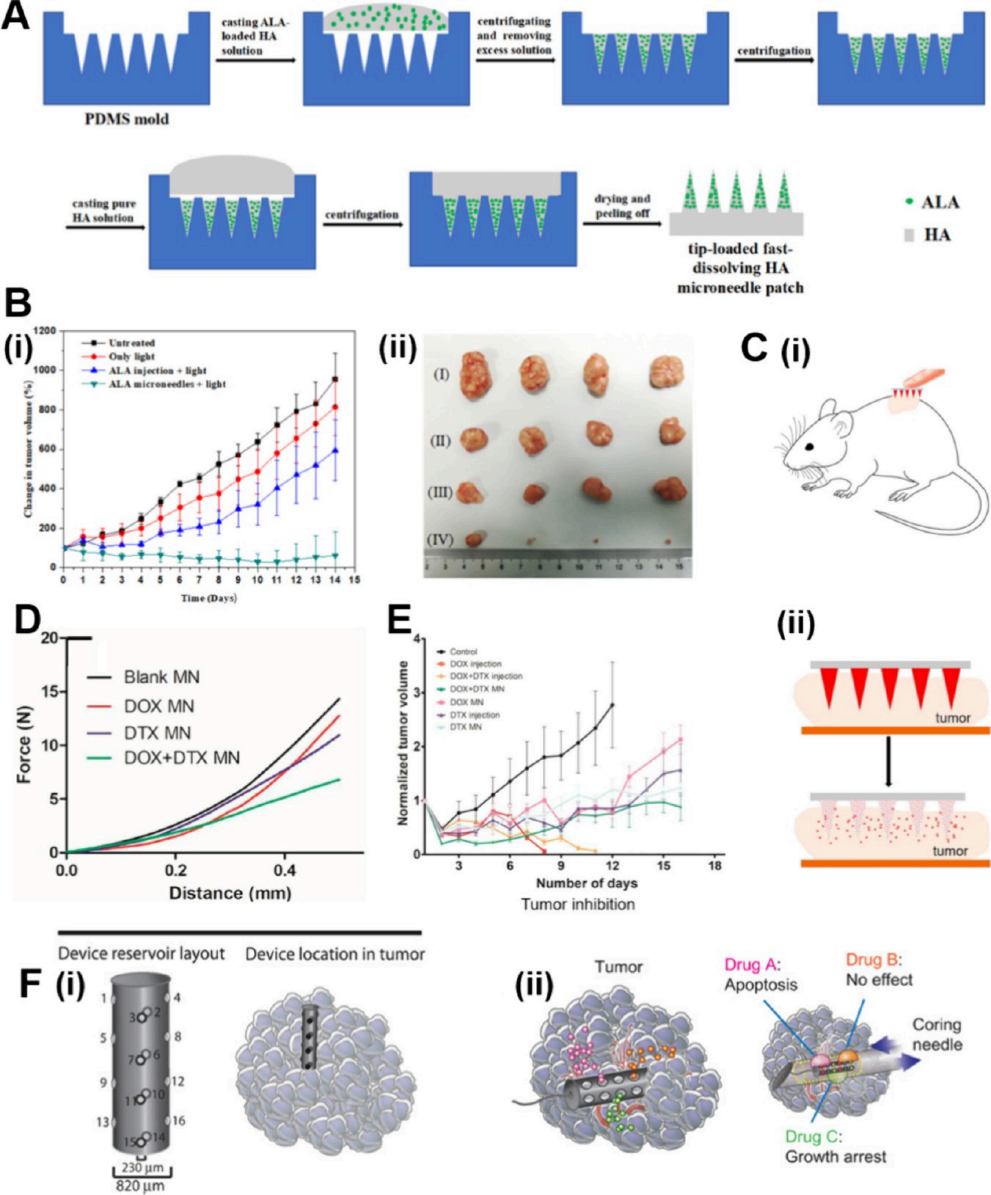
Preparation and antitumor capabilities of common microneedle patches. (A) Schematic illustration of the process to fabricate the tip-loaded fast-dissolving HA microneedle patch. Abbreviations: ALA, 5-Aminolevulinic acid; HA, hyaluronate. (B) (i) Tumor growth curves of different groups of tumor-bearing mice after treatment. (ii) Weight of excised tumor of each group after 14 days’ treatment. Each data point represents mean ± standard deviation (*n* = 4). [Reprinted with permission from ref 154. Copyright 2018, Elsevier B.V.] (C) (i) Schematic illustration of the microneedle application to tumor-bearing mice. (ii) Schematic illustration of the microneedle array dissolving to releasing drug. (D) Displacement graph for assessing the compression force for various microneedle patches. Abbreviations: DTX, docetaxel. (E) Normalized tumor volumes of mice in different treatment groups during the study. Data presented as Mean ± error (*n* = 3–6). [Reproduced with permission from ref 140. Copyright 2018, Elsevier] (F) (i) Panel of 16 distinct reservoirs from a single device. (ii) The device is implanted by needle directly into tissue, and drugs diffuse from device reservoirs into confined regions of tumor. [Reproduced with permission from ref 141. Copyright 2015, The American Association for the Advancement of Science].

The local application of microneedle-based systems enhances drug penetration into deeper tumor regions while minimizing the leakage of drugs into adjacent tissues, thereby mitigating side effects.^150–152^ Recent studies have demonstrated the utility of microneedle patches as a novel synergistic system in conjunction with various therapies, facilitating the repetitive delivery of various therapeutic factors to superficial tumors, thereby improving cancer treatment.^153^ Zhao et al.^154^ fabricated a hyaluronic acid–based microneedle patch loaded with 5-aminolevulinic acid. This compound is converted into protoporphyrin in mitochondria to serve as a photosensitizer (PS) that mediates the production of highly reactive oxygen species (ROS) under light irradiation, thus providing an effective approach for tumor treatment. The microneedle patch was created by depositing a mixture of 5-aminolevulinic acid and hyaluronic acid. Subsequent in vivo experiments demonstrated its ability to markedly improve drug utilization and effectively inhibit and eradicate tumors (Figure 4 B). Bhatnagar et al.^140^ developed a polyvinylpyrrolidone/poly­(vinyl alcohol) microneedle patch loaded with DOX and docetaxel for the combination treatment of breast cancer (Figure 4 C). These microneedle patches exhibited compressive strength greater than 5 N and successfully punctured the skin of mice (Figure 4 D). Furthermore, in vivo studies showed that this combination of DOX and docetaxel is more efficacious than a single agent in effectively inhibiting tumors (Figure 4 E). Jonas et al.^141^ developed a microneedle featuring several drug reservoirs on the cylindrical surface of the device with the capacity to accommodate up to 16 reservoirs, each of which is independent of the other (Figure 4 F). This design allows for the modification of drug formulations to target specific tumors. Drug diffusion by these microneedles can be controlled by adjusting the size of the reservoirs. Additionally, they can be implanted into tumors using a biopsy needle to release various drugs.

Microneedle-based systems offer several advantages, including compact size, minimally invasive nature, user-friendliness, superior skin penetration capabilities, and the ability to facilitate transdermal drug absorption. Consequently, microneedle patches are highly effective in delivering drugs to superficial tumors through the skin, granting them a significant advantage in targeting superficial tumors. Moreover, they enable a substantial reduction in drug dosage, effectively improving therapeutic efficacy and diminishing drug-related side effects.^31,155–157^ However, the application of microneedle patches for percutaneous tumor treatment may be compromised by exposure to external pathogens and may not yield the desired results when treating deep-seated tumors.

### 3.7 Others

Various forms can serve as LDDSs. In addition to the ones mentioned earlier, other options include wafers, NPs, and liposomes, which we will briefly describe below.

Wafers are a category of biodegradable polymers containing drugs, with the most notable example being the Gariadel Wafer.^158^ The blood-brain barrier has been shown to block the delivery of most drugs from the bloodstream to the brain. Hence, novel technologies need to be explored for circumventing the blood-brain barrier.^159^ One controversial yet intriguing approach involves directly implanting drugs into the central nervous system. For example, drug-carrying polymer systems can be implanted directly into the brain to treat brain disorders.^160^ One exceptionally successful system is the Gariadel Wafer, which was developed in the early 1980s and approved by the Food and Drug Administration (FDA) in 1996. Its performance was evaluated in multiple clinical trials.^161–163^ The Gariadel Wafer is a carmustine-loaded biodegradable polyhydride wafer. It has been primarily employed in the treatment of malignant gliomas, such as glioblastoma multiforme, and can be implanted into the brain after surgical removal of the tumor to facilitate controlled release of carmustine to treat gliomas that cannot be completely removed, thereby bypassing the blood-brain barrier.^38,164,165^

NPs and liposomes can be used in various ways, either as carriers for drug delivery or as drugs for tumor therapy.^166^ They can also be combined with other macroscopic LDDSs, such as hydrogels, to create nanohydrogel composites, thereby enhancing their performance.^167^ Therefore, NPs and liposomes can be classified as drugs, SDDSs, or LDDSs depending on their application.^168^ Here, we provide a brief introduction to the portion of these entities that pertain to LDDSs.

Currently, NPs utilized in LDDSs mostly serve as transport carriers for traditional drugs. Gold NPs (GNPs) are widely favored by researchers due to their facile processability into various shapes. Spherical GNPs, gold nanorods, gold nanoshells, and gold nanoclusters are commonly used, with spherical GNPs being the preferred choice because of their relatively simple production and modifiability of specifications and properties. However, this straightforward approach to encapsulating chemotherapeutic drugs in NPs has not yielded significant improvements in drug utilization. Thus, attention has shifted toward exploring novel NPs.^169–171^ For example, Rynda-Apple et al.^172^ found that certain virus-like particles (VLPs) exhibit inherent immunogenicity. Additionally, a few researchers have suggested that VLPs induce the generation of neutralizing antibodies against viral coat proteins.^173^ Other researchers have reported that VLPs can also elicit immunomodulation. For instance, Lizotte et al.^174^ found that self-assembling virus-like NPs derived from cowpea mosaic virus (CPMV) can induce potent systemic antitumor immunity and inhibit B16F10 melanoma. These CPMV NPs are 30 nm icosahedrons composed of 60 shell protein units. They can be used to encapsulate drugs, serving as drug transport systems, owing to their hollow, stable, nontoxic, and highly scalable properties. Subsequent experiments have demonstrated that CPMV NPs also induce significant systemic antitumor immunity and exert therapeutic effects in various tumor models, such as ovarian, colon, and breast tumor models.

Liposomes offer the advantages of biocompatibility, self-assembly capability, high drug loading capacity, etc. They can be classified according to their morphology and size into three types: multilamellar vesicles (0.1–20 μm), large unilamellar vesicles (0.1–1 μm), and small unilamellar vesicles (25–100 nm).^175^ Their fabrication process is not comprehensively described here but can be found in the works of Perrett et al.,^176^ Hope et al.,^177^ Szoka et al.,^178^ and Bangham et al.^179^ Liposomes have diverse applications. For example, Li et al.^180^ reported a novel and efficient DNA hybridization recognition-mediated method for targeted liposome drug delivery. They inserted lipid-terminated DNA into cell membranes and drug-encapsulated liposomes through a simple mixing process. When the DNA on the cell membrane complemented the targeted DNA sequences linked to liposomes, it led to the accumulation of liposomes around the cell. This enhanced the cellular uptake of liposomes, facilitating efficient delivery and internalization of the loaded drug. Koshkina et al.^181^ and Garbuzenko et al.^182^ investigated the effects of trans-tracheal administration of liposomes loaded with PTX and DOX, respectively. The results showed that transtracheal administration via liposomes outperformed systemic administration, resulting in slower clearance and a higher concentration of the drug in the lungs, along with reduced toxic side effects on normal tissues.

Each LDDS offers distinct advantages in addressing the key issues in oncology treatment and can be applied in diverse scenarios. However, they are not suitable for all oncology treatment scenarios. As a result, many novel LDDSs are under development, but in practice, the boundaries between these various delivery systems are not clear-cut, as they exhibit similarities or can be integrated or converted into one another. For example, linear fibers can be converted into two-dimensional fibrous mats or 3D scaffolds for tumor therapy, while silk can be converted into various forms, such as hydrogels, sponges, films, scaffolds, and NPs, through processing after being broken down into silk proteins.^36^ Therefore, when discussing LDDSs for tumor treatment, our focus lies not on the form of delivery systems but rather on the methods and mechanisms of LDDS-mediated tumor therapies.

## 4 Single Cancer Therapy Based on LDDSs

Taking chemotherapy as an example, traditional chemotherapy delivery methods face challenges in effectively locally targeting tumors for precise treatment due to their broad range of action, short duration of single administration of chemotherapy drugs, inadequate local accumulation in the tumor, low therapeutic efficiency, etc.^183^

To achieve an effective local drug concentration in the tumor, drug dosages are often elevated, which exacerbates toxic side effects and increases the number of adverse reactions. Zhang et al.^184^ developed poly­(ethylene glycol)-DOX-curcumin (PEG-DOX-Cur) NPs, which increased the accumulation of DOX in tumor tissues by nearly 4-fold compared to intravenous DOX injection at an equivalent dose. Furthermore, the in vivo experiments showed that the tumor volume in the PEG-DOX-Cur NP group was nearly half that in the DOX/Cur group, while body weight was more than double. Additionally, Han et al.^185^ conducted animal experiments involving chitosan-loaded DOX (CH-DOX), revealing that the tumor volume in the intratumorally injected CH-DOX group was 1/8 of the tumor volume in the intravenously injected DOX group. Thus, the aforementioned issues can be more effectively addressed using LDDSs, which can reduce chemotherapeutic drug quantities, improve single-dose duration of action and therapeutic efficiency, reduce the toxicity and adverse effects of drugs, and provide precise local targeting of the tumor. Therefore, LDDS-mediated tumor therapies confer significant advantages, as described below. Figure 5 illustrates common therapies and LDDS.

**Figure 5:**
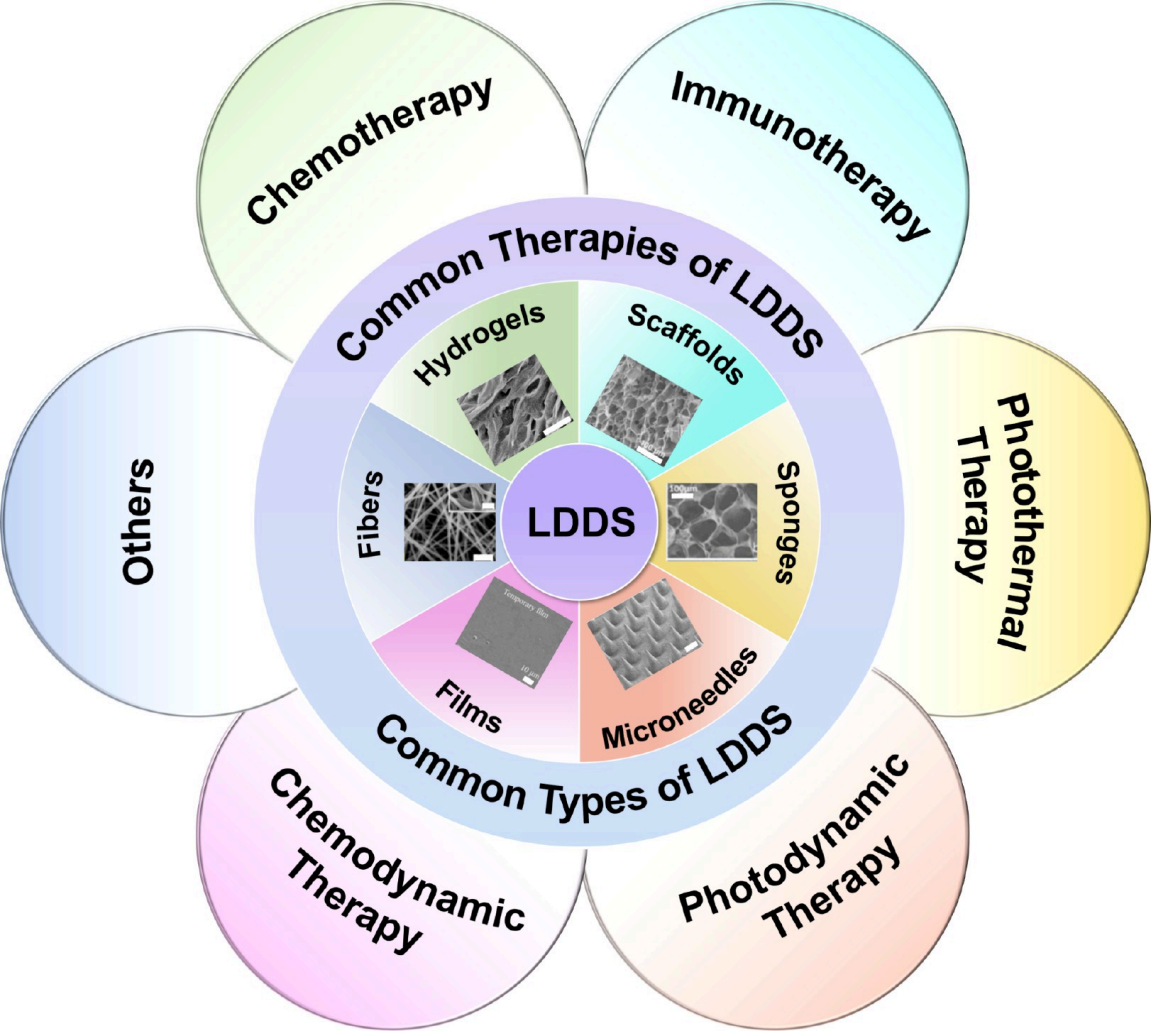
Schematic illustration of common therapies and LDDS. Images are reproduced with permission from the cited articles: microcosmic fibers (Scale bar below: 5 μm, scale bar above: 2 μm),^28^ [Reproduced with permission from ref 28. Available under a CC BY license. Copyright 2020 Tao Chen et al.] microcosmic hydrogels (Scale bar: 10 μm),^30^ [Reproduced with permission from ref 30. Copyright 2024, Bentham Science Publishers B.V.] microcosmic scaffolds (Scale bar: 100 μm),^29^ [Reproduced from ref 29. Copyright 2022 American Chemical Society] microcosmic sponges (Scale bar: 100 μm),^34^ [Reproduced from ref 34. Copyright 2022 American Chemical Society] microcosmic microneedles (Scale bar: 200 μm),^32^ [Reproduced from ref 32. Copyright 2022 American Chemical Society] microcosmic films (Scale bar: 10 μm).^35^ [Reproduced with permission from ref 35. Available under a CC BY license. Copyright 2022 Denghang Xie et al.].

### 4.1 Chemotherapy

Chemotherapy is a traditional tumor treatment method, often administered orally or intravenously.^186^ Chemotherapeutic drugs reaches the tumor site after systemic circulation, eliminating tumor cells, and can also inhibit tumor metastasis and improve local eradication of primary tumors.^187,188^ However, chemotherapeutic agents can cause damage to normal cells, resulting in corresponding toxic side effects.^189^ Similar to radiation therapy, these toxic side effects caused by chemotherapeutic agents remain one of the significant limitation in the wider application of chemotherapy in the treatment of tumors.^190,191^ Therefore, there is a need to improve the bioavailability of drugs in the tumor region while minimizing their distribution to surrounding tissues. This approach aims to lower both the dosage of chemotherapeutic drugs used and their concentration in normal tissues, thereby reducing the extent and severity of toxic side effects.^192^

Local chemotherapy involves delivering anticancer drugs directly to the affected organ, ensuring higher drug concentrations at the tumor site compared to other sites. Utilizing NPs for the delivery of oncology chemotherapeutic drugs has proven to be effective and safe across a wide spectrum of cancers.^193–195^ Biocompatible and biodegradable materials are commonly employed in the preparation of NPs, which can be loaded with chemotherapeutic drugs to facilitate targeted and controlled drug delivery ( Figure 6).^196–199^ Additionally, NPs possess a slow-release property that enables chemotherapeutic drugs to maintain an effective concentration locally at the tumor site for an extended duration.^200,201^ For example, Ma et al.^202^ prepared PTX-loaded PCL/F68 NPs characterized by spherical morphology with rough and porous surfaces. In a mouse breast cancer model, despite using an equivalent dose of PTX, a single intratumoral injection of PTX-loaded PCL/F68 NPs demonstrated greater efficacy in halting tumor progression compared to multiple intraperitoneal injections of PTX. Therefore, employing LDDSs for chemotherapeutic drug delivery confines the treatment to the tumor site, sustains the effective lethal concentration over an extended period, and minimizes systemic dispersion of the chemotherapeutic drug, thereby minimizing toxic and adverse effects.^203^

**Figure 6:**
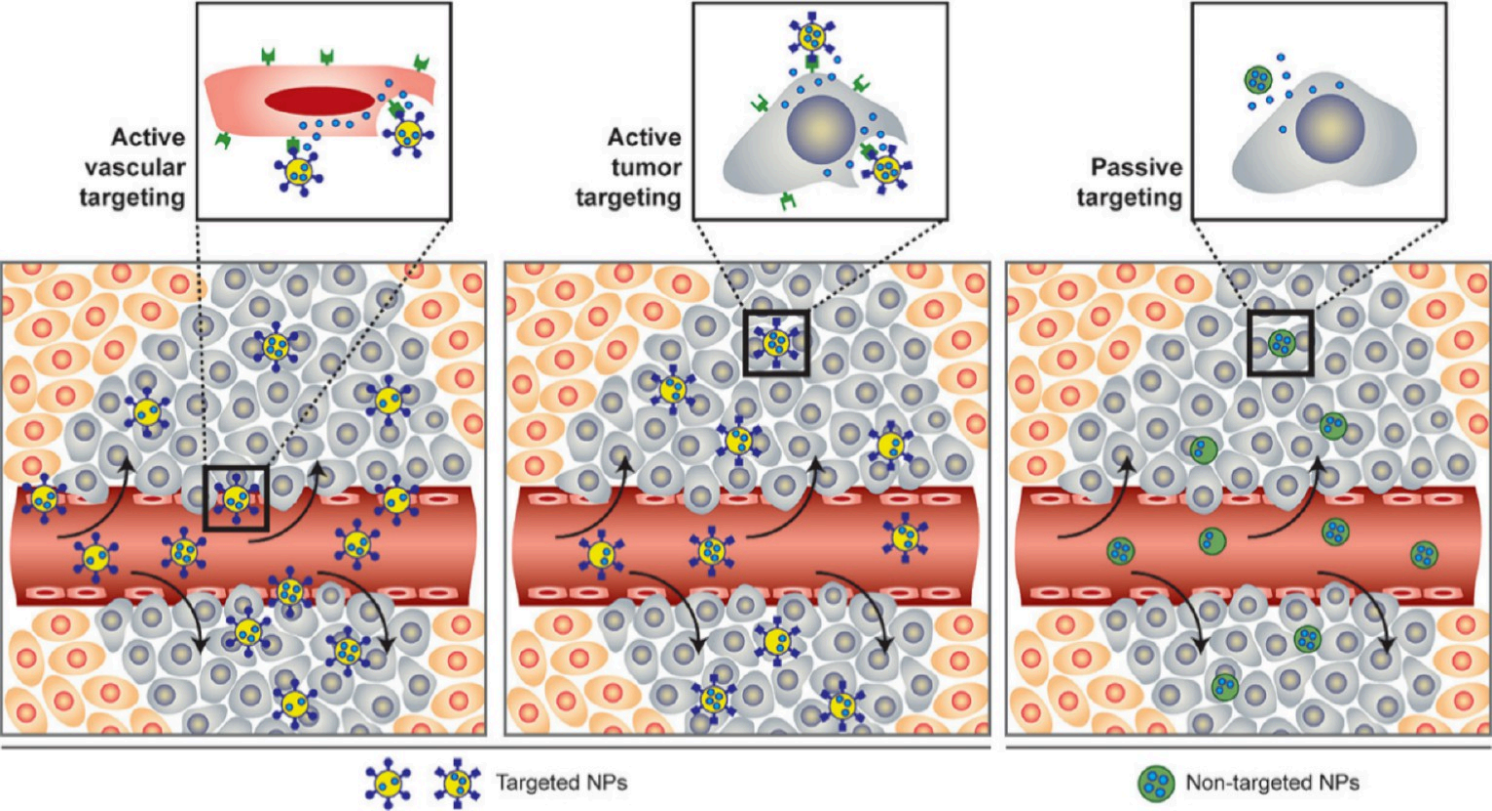
Passive vs active targeting of NPs. (Right) Particles tend to passively extravasate through the leaky vasculature, which is characteristic of solid tumors and inflamed tissue, and preferentially accumulate through the EPR effect. In this case, the drug may be released in the extracellular matrix and diffuse throughout the tissue for bioactivity. (Middle) Once particles have extravasated in the target tissue, the presence of ligands on the particle surface can result in active targeting of particles to receptors that are present on target cell or tissue resulting in enhanced accumulation and cell uptake through receptor-mediated endocytosis. This process, referred to as “active targeting”, can enhance the therapeutic efficacy of drugs, especially those which do not readily permeate the cell membrane and require an intracellular site of action for bioactivity. (Left) The particles can be engineered for vascular targeting by incorporating ligands that bind to endothelial cell-surface receptors. While the presence of leaky vasculature is not required for vascular targeting, when present as is the case in tumors and inflamed, this strategy may potentially work synergistically for drug delivery to target both the vascular tissue and target cells within the diseased tissue for enhanced therapeutic. [Reproduced from ref 198. Copyright 2009 American Chemical Society].

### 4.2 Immunotherapy

Under normal circumstances, the body’s immune system can recognize and eliminate tumor cells. However, tumor cells have the capability to release immunosuppressive molecules, allowing them to evade immune surveillance.^204^ Consequently, methods to activate the body’s antitumor immune response have attracted much attention.^205,206^ Simultaneously, the immunosuppressive potential of the TME can lead to the infiltration of effector lymphocytes and functional impairment, rendering the tumor ″cold″ and substantially diminishing the antitumor immune response.^207^ Thus, the actual efficacy of tumor immunotherapy is much lower than expected (Figure 7).^208^ The systemic administration of immunotherapy must contend with the nonspecific distribution of immunotherapeutic drugs and necessitate a cycle to stimulate the immune system, involving various immune factors and bodily processes. This approach is prone to causing systemic immune dysregulation and other adverse effects.^209,210^ Therefore, devising a rational treatment strategy to transform tumors into a ″hot″ state while mitigating systemic treatment-related effects is the key to improving the effectiveness and applicability of antitumor immunotherapy.^211^

**Figure 7:**
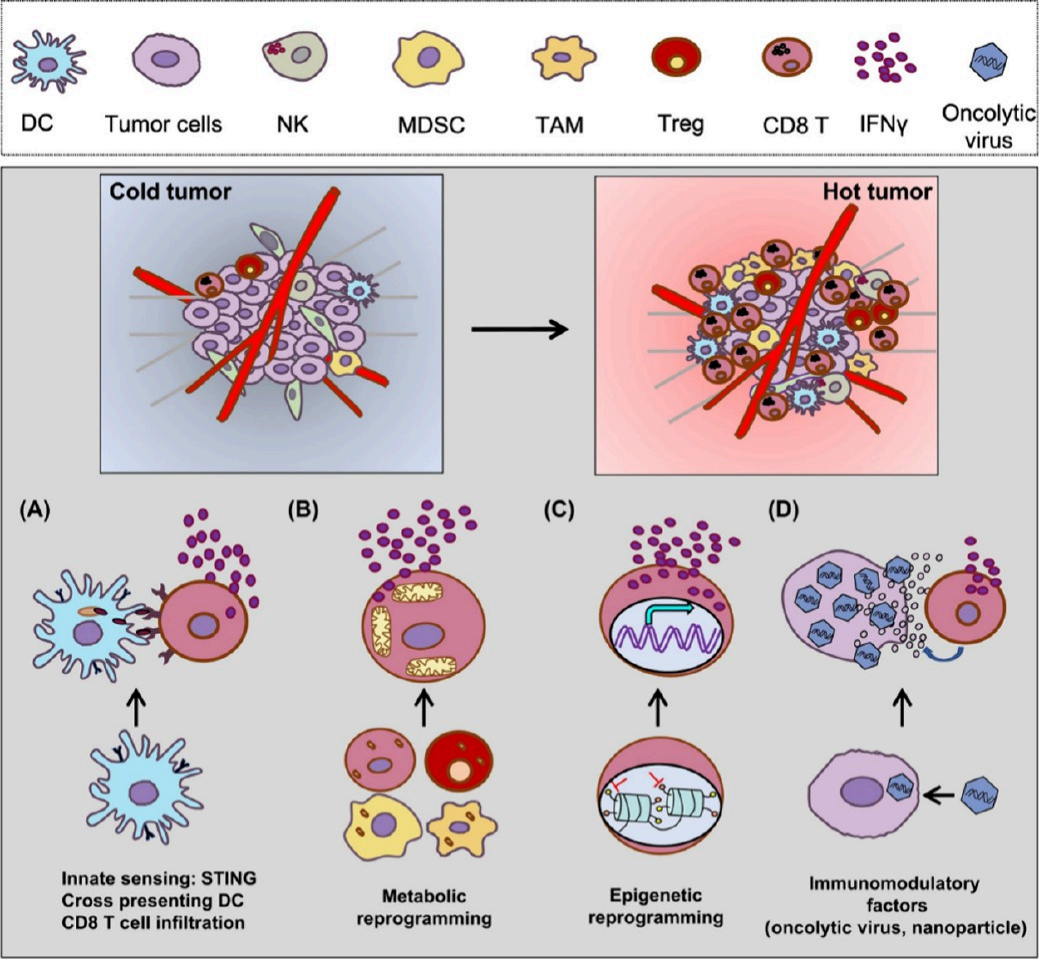
Several key strategies that target multiple steps of the cancer immunity cycle. (A) Induced activation of the innate immune sensing system with STING agonists or boosting cross presenting DCs to promote tumor-antigen-specific T cell trafficking or function within the TME. (B) Targeting cellular metabolism and certain metabolites within the TME to reduce immunosuppressive Tregs, MDSCs, or TAMs, or to generate metabolically fit T cells with better mitochondrial activity to protect against the tumor. (C) Targeting epigenetic modulators to either promote immunogenicity of tumor cells or to re-educate TAMs, MDSCs, or Tregs for the support of T cell effector functions. (D) Creating an inflamed TME via oncolytic virus or nanoparticle delivery of key immunomodulatory factors. Abbreviations: DC, dendritic cell; IFN, interferon; MDSC, myeloid-derived suppressor cell; NK, natural killer; TAM, tumor-associated macrophage; Treg, regulatory T cell. [Reproduced with permission from ref 208. Copyright 2020, Elsevier Inc.].

Therapeutic approaches initially focused on activating the body’s own tumor immunity, such as certain 3D scaffolds that recruit and modulate host immune cells, involving the sustained release of inflammatory signals and adjuvants for immunomodulation. These approaches significantly delay tumor growth.^212^ Exogenous immunopharmaceuticals have also been employed, such as microneedle machine systems for the delivery of immunopharmaceuticals. These systems also significantly inhibit tumor growth and improve the survival rate of mice.^213^ For example, Wang et al.^32^ deposited dextran NPs loaded with antiprogrammed death 1 (aPD-1) antibodies and glucose oxidase on modified hyaluronic acid microneedles. When these microneedles penetrated mouse skin, glucose oxidase catalyzed the conversion of glucose to gluconic acid, promoting the release of aPD-1 from the NPs. This process effectively stimulated the infiltration of CD8+ T cells into tumor tissues in a B16F10 mouse melanoma model, resulting in significant tumor growth inhibition and improved mouse survival rates. Therefore, tumor immunotherapy using LDDSs can enhance the body’s immune and tumor immune responses. They also weaken the tumor’s natural protective barrier and can even exert a direct suppressing effect on the tumor. Additionally, they circumvent the side effects associated with traditional immunotherapy, such as systemic immune dysregulation.^214^ Thus, local tumor immunotherapy holds promise as a targeted drug delivery method that minimizes systemic toxicity and improves therapeutic efficiency.^215^

### 4.3 Photothermal Therapy

PTT is a treatment method that involves the injection of materials with high photothermal conversion efficiency into the tumor tissue region. Subsequently, these materials are irradiated by an external light source, typically near-infrared (NIR) light. This irradiation results in the conversion of light energy into heat energy, ultimately eliminating tumor cells. Typically, NIR encompasses NIR-I (650–1000 nm) and NIR-II (1000–1350 nm). NIR-II exhibits superior tissue penetration compared to NIR-I, resulting in enhanced photoavailability during in vivo experiments. NIR light-responsive PTT has certain potential advantages, such as simplicity of operation, high tumor thermal ablation effect, and minimal chemoresistance.^216–218^ Currently, PTT has found application in the treatment of cancer; however, the vast majority of photothermal agents lack targeting. When administered intravenously, only a small portion of these agents eventually accumulate in tumor tissues, resulting in severe toxic side effects.^219–221^ Therefore, delivery of photothermal agents via LDDSs is necessary to improve efficacy and mitigate systemic toxicity.

Currently, commonly used photothermal agents include various nanomaterials such as gold NPs and composite photothermal materials.^222–227^ Simultaneously, various excellent photothermal therapeutics are in development. For instance, Fu et al.^228^ prepared Cu_2_MnS_2_ nanosheets, which were subsequently incorporated into Pluronic F127 hydrogels. The Cu_2_MnS_2_ nanosheets efficiently convert light energy into heat energy, achieving a maximum in vivo temperature close to 90 °C. Additionally, they demonstrated a stable heating curve, indicating an obvious PTT effect against tumors. However, although localized PTT via LDDSs can mitigate the toxic side effects associated with photothermal agents to the greatest extent and restrict their action to the tumor site, maximizing therapeutic efficiency, it typically fails to limit distant tumor recurrence and metastasis. Thus, it is necessary to complement this approach with additional therapies.^80^

### 4.4 Photodynamic Therapy

PDT involves activating the PS using specific wavelengths of light. This activation leads to the generation of ROS through a photodynamic reaction, resulting in the eradication of tumor cells.^229,230^ ROS can cause oxidative damage to endogenous biomolecules (proteins, DNA, etc.), triggering local inflammation and ischemia and inducing necrosis and apoptosis in tumor cells. These ROS primarily include O^2–^, −OH, and H_2_O_2_.^231–235^ The efficacy of PDT depends on three components: the light source, the PS, and O_2._ ^236–238^ In the process of PDT, initially, a PS such as porphyrin is typically administered to the tumor site and then irradiated with specific wavelengths of light, allowing the PS to absorb photons and transitions from the ground state to the excited state.^239^ This transition releases energy into the surrounding environment, leading to its conversion to ROS and ultimately eradicating tumor cells.^240^

However, in conventional PDT, most PSs, such as porphyrin, phthalocyanine, and phenothiazine, have short half-lives in the bloodstream, lack stability, and lack tumor targeting capability. This can lead to the underutilization of PSs and unwanted side effects. Therefore, it is necessary to facilitate the localized accumulation of PSs in tumors using LDDSs.^241^ Luo et al.^242^ employed methoxy poly­(ethylene glycol)-polylactide copolymer (mPEG–PDLLA) and Pluronic F127 to prepare a temperature-sensitive hydrogel, which was then loaded with pyropheophorbide a (PPa) and a two-photon absorption compound (T1). PPa is considered a more superior second-generation PS, while T1 is simply capable of effectively absorbing near-infrared light, aiding in PDT. Subsequent experiments showed that this hydrogel effectively localizes both PDT reagents in the tumor and prolongs their intratumor retention time, even effectively inhibiting tumors located beneath 1 cm-thick muscle tissue. Thus, LDDSs enhances the tumor targeting of PSs, facilitate their accumulation in tumors, and prolong their intratumor retention time, markedly reducing the impact of PS on normal tissues and enabling efficient tumor treatment with PDT. This broadens the prospects and potential of PDT as a novel oncology therapy.^243^ However, owing to the limited tissue penetration of NIR light, PDT is primarily applicable in the treatment of superficial tumors, such as melanoma.^244,245^

### 4.5 Chemodynamic Therapy

Chemodynamic therapy (CDT), an emerging field in tumor therapy in recent years, involves harnessing endogenous and spontaneous energy within the tumor to selectively eliminate tumor cells through the design of nanomedicines tailored to the TME, obviating the need for external energy sources.^246^ The iron-induced intratumor Fenton reaction serves as an illustrative example. In the acidic milieu of the TME, Fe^2+^ can react with a surplus of H_2_O_2_ and decompose it into cytotoxic hydroxyl radicals (−OH), responsible for eliminating tumor cells.^247^ Thus, a pivotal determinant of CDT efficacy lies in the local levels of metal ions and H_2_O_2_ in the tumor. Therefore, effective metal ion delivery and high levels of H_2_O_2_ are imperative prerequisites for successful CDT outcomes.^248^

Therefore, Wang et al.^249^ reduced ferrous ions in situ within mesoporous silica NPs (MSN) mesopores to prepare FeMSN for the first time. Subsequently, they immobilized FeMSN onto the surface of electrospun PCL-gelatin fibers (PG fibers) through electrostatic interactions for the purpose of delivering ferric ions (Figure 8 A, 8 B). They demonstrated that under the acidic conditions of the TME, FeMSN is released from the composite fibers and effectively taken up by 4T1 cancer cells. This uptake induces the Fenton reaction, resulting in the production of −OH, thereby exhibiting a significant tumor cell-killing effect. Furthermore, the FeMSN@PG composite fibers served as a reservoir, allowing for the continuous and efficient consumption of endogenous substances in the TME for efficient CDT treatment. These composite fibers demonstrated longer-lasting and more potent anticancer effects compared with the direct intratumoral injection of FeMSN. Although CDT has found widespread applications as a therapeutic modality, its effectiveness diminishes considerably when tumors experience depletion in endogenous substances and energy. This limitation arises from the inherent constraints on the available resources within the tumor microenvironment. Consequently, the maintenance of sustained CDT therapy is a crucial consideration.^250,251^

**Figure 8:**
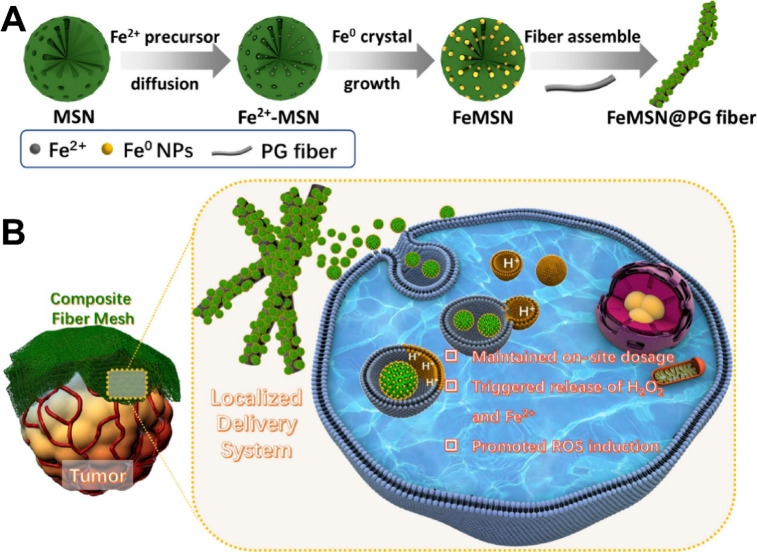
Synthesis and Therapeutic Schematic of FeMSN@PG Fibers. (A) Schematic illustration of the synthesis of Fe^0^ nanocrystal embedded MSN (FeMSN). (B) Schematic illustration of FeMSN@PG fibers for localized CDT approach. [Reproduced with permission from ref 249. Copyright 2020, Elsevier B.V.].

### 4.6 Others

The aforementioned therapies are some commonly utilized approaches, yet they do not encompass the entire spectrum of treatments achievable through LDDSs for tumors. Other options are sonodynamic therapy (SDT), starvation therapy (ST), gene therapy (GET), metabolic therapy (MT), gas therapy (GAT), and more.

SDT, an emerging tumor treatment, relies on a sonic sensitizer in conjunction with ultrasound waves to induce cell damage, similar to PDT. SDT generally employs low-intensity ultrasound radiation to activate the acoustic sensitizer. Upon the acoustic sensitizer’s return to its ground state, the released energy can transfer to surrounding oxygen, leading to the generation of substantial amounts of ROS and ultimately triggering apoptosis. A significant advantage of SDT over PDT is its precise ultrasound focusing capability, with the potential to penetrate soft tissues up to tens of centimeters. Consequently, it is considered by numerous researchers as a promising novel therapeutic approach that has demonstrated promising anticancer outcomes in both in vitro and in vivo studies.^252^

High cellular metabolism and substantial nutrient depletion are crucial physiological characteristics of tumors. ST exploits these characteristics, depriving them of glucose, oxygen, and/or other key nutrients through blood supply obstruction. This, in turn, triggers tumor cell apoptosis due to the lack of nutrients.^253–255^ Tumor therapeutic strategies involving ST have been extensively studied and are widely recognized as an attractive approach to cancer treatment.^256^

While MT, refers to an approach that targets and reprograms the metabolic pathways of tumor cells, particularly focusing on essential nutrients or metabolites that are heavily utilized by cancer cells for growth, survival, and metastasis.^257,258^ By disrupting normal tumor metabolism, metabolic therapy creates opportunities for immune cells and other stromal components to intervene in cancer progression.^259^ Therefore, as an emerging tumor therapy, it not only inhibits tumor growth but also enhances the body’s antitumor immunity, offering broad application prospects, particularly in combination with other therapies.

GET involves transferring therapeutically active genetic material, such as DNA and RNA, into cells to rectify defective gene expression by introducing an accurate copy of the flawed gene, thereby treating the disease. Since cancer arises from faulty gene mutations, GET can inhibit cancer at its source and may emerge as a promising treatment modality for various cancer types. Currently, GET for cancer primarily targets the stimulation of protective immune responses against tumors, substitution of mutated tumor suppressor genes, inactivation of oncogenes, induction of suicide genes, etc.^260^

The treatment of tumors through the localized application of specific gases known as GAT and has emerged as a novel and effective therapy with minimal side effects. To date, three primary gas molecules have been employed in this research field: nitric oxide (NO), carbon monoxide (CO), and hydrogen sulfide (H_2_S). These are naturally occurring gas signaling molecules in mammals and play crucial roles in various physiological and pathophysiological processes. Recent studies have demonstrated that these signaling molecules may selectively inhibit cancer cells by inducing cell cycle arrest, regulating microRNAs, causing mitochondrial damage, etc.^261^

The utilization of LDDSs can mitigate toxic side effects and adverse reactions associated with various drugs, thereby enhancing therapeutic efficiency compared with conventional drug delivery methods. However, the utilization of LDDSs concentrates most chemotherapeutic drugs in the tumor, rendering them ineffective against undetected microtumor foci and metastatic tumor cells, which represents an inherent challenge when employing LDDSs as a standalone approach in oncological treatment. Simultaneously, although LDDSs provide a superior platform for demonstrating the efficacy of certain novel therapeutic approaches, such as CDT and SDT, we contend that their true value lies in complementing to maximize their individual contributions. This approach will enable the achievement of local multifunctional synergistic oncology treatments, optimizing overall efficacy. Thus, we will introduce them below.

## 5 Multifunctional Synergistic Therapies Based on LDDSs

Single tumor therapies have certain limitations and are gradually falling short of meeting people’s need for high-quality tumor treatments. Thus, researchers are increasingly turning their attention to combination tumor therapies.^262^ Multifunctional synergistic oncology therapies can address the limitations of single therapies and even enhance treatment outcomes. As a result, multifunctional synergistic oncology therapies are expected to emerge as the predominant research direction in the future.^263–266^ However, multifunctional synergistic oncology therapies involving traditional drug delivery methods present several challenges, including cumbersome drug delivery procedures, high drug dosages, many toxic side effects, and substantial treatment difficulties.^267^

LDDSs, such as hydrogels, scaffolds, sponges, and films, exhibit extremely high loading capacities, implying that they can simultaneously accommodate multiple therapeutic agents, mitigating the challenges associated with multifunctional synergistic oncology therapies when employing conventional drug delivery methods.^41,268,269^ Among these, responsive LDDSs are preferred by researchers due to their ability to facilitate controlled drug release and tailored tumor therapies, thereby enhancing drug utilization efficiency and therapeutic efficacy, ultimately resulting in improved performance.^270^

Among the various controllable strategies, the photothermal response is the most commonly employed due to its capacity to elevate local temperatures through simple irradiation. This, in turn, expedites the desired reaction and facilitates the photothermal ablation of tumors.^271^ However, most photothermal agents are not targeted, and their administration through conventional means can lead to their significant accumulation in healthy tissues while maintaining an effective concentration in the tumor. Consequently, this can result in severe toxic side effects.^272^ In contrast, the utilization of photothermoresponsive LDDSs loaded with photothermal agents not only minimizes toxic side effects to the greatest extent possible but also activates a predetermined function when light energy is converted into heat by the photothermal agent, thereby fully exploiting the synergy between different therapies to enhance oncological efficacy.^261,262^ Therefore, here we focus on combination therapies mediated by photothermal responsiveness.

### 5.1 Photothermal-Chemotherapy

Various anticancer chemotherapeutic drugs, such as DOX and PTX, have been successfully applied in the clinic. However, owing to tumor drug resistance and inadequate local penetration, chemotherapy alone typically cannot eliminate tumors completely and results in systemic toxic side effects.^273^ In contrast, the combination of localized PTT and chemotherapy can be used for photothermal ablation of tumors alongside improved drug uptake by tumor cells, thereby enhancing the efficacy of chemotherapy.

Rong et al.^274^ utilized mesoporous polydopamine (MPDA) NPs as a photothermal agent. They loaded DOX onto MPDA NPs through π-π stacking, creating DOX@MPDA NPs as the main body for tumor therapy. Subsequently, these NPs were loaded into a composite hydrogel comprising oxidized hyaluronic acid (OHA) and hydroxypropyl chitosan (HPCS) (Figure 9 A). This injectable hydrogel exhibits superior self-healing properties. Notably, the authors believed that PTT could trigger the production of inflammatory factors by tumor necrotic cells, promoting the growth of tumor cells and diminishing therapeutic efficacy. Thus, they introduced curcumin (CUR) modified with amino-modified β-cyclodextrin to improve the solubility of CUR and facilitate post-treatment anti-inflammation. When 0.4 wt % MPDA NPs were irradiated with 1 W/cm^2^ NIR, the irradiation site’s temperature reached 65 °C in 5 min (Figure 9 B). Additionally, the photothermal light elevated the surface temperature of the MPDA medium and increased molecular motion. Consequently, this reduced π-π stacking between MPDA medium and DOX accelerated the release of DOX. In vitro experiments showed that DOX-MPDA&CUR@Gel alone exhibited minimal impact on tumor cells (approximately 75% activity), and NIR irradiation alone had negligible effects. However, irradiating the two combinations for 4 and 6 min significantly decreased cellular activity to 30% and 20%, indicating that this composite hydrogel exerts a superior inhibitory effect on the proliferation of tumor cells (Figure 9 C). The subcutaneous tumor temperature of the intratumorally injected composite hydrogel, which contained MPDA NPs, increased to 57 °C within 5 min in vivo (Figure 9 D). And there was no significant difference in the body weight change of each group throughout the whole treatment (Figure 9 E). Concurrently, the tumor growth rate in the DOX-MPDA&CUR@Gel+NIR group was significantly lower than that in the other groups, resulting in the smallest tumor volume at 14 days, representing only approximately 20% of the volume observed in the PBS-injected group (Figure 9 F). Furthermore, cytokine levels were significantly lower in the irradiated group containing CUR and MPDA nanoparticles, suggesting that CUR within the composite gel reduced PTT-induced inflammation (Figure 9 G). These findings suggest that the DOX-MPDA&CUR@Gel, which integrates chemotherapy and PTT, exhibits significant advantages in tumor treatment.

**Figure 9:**
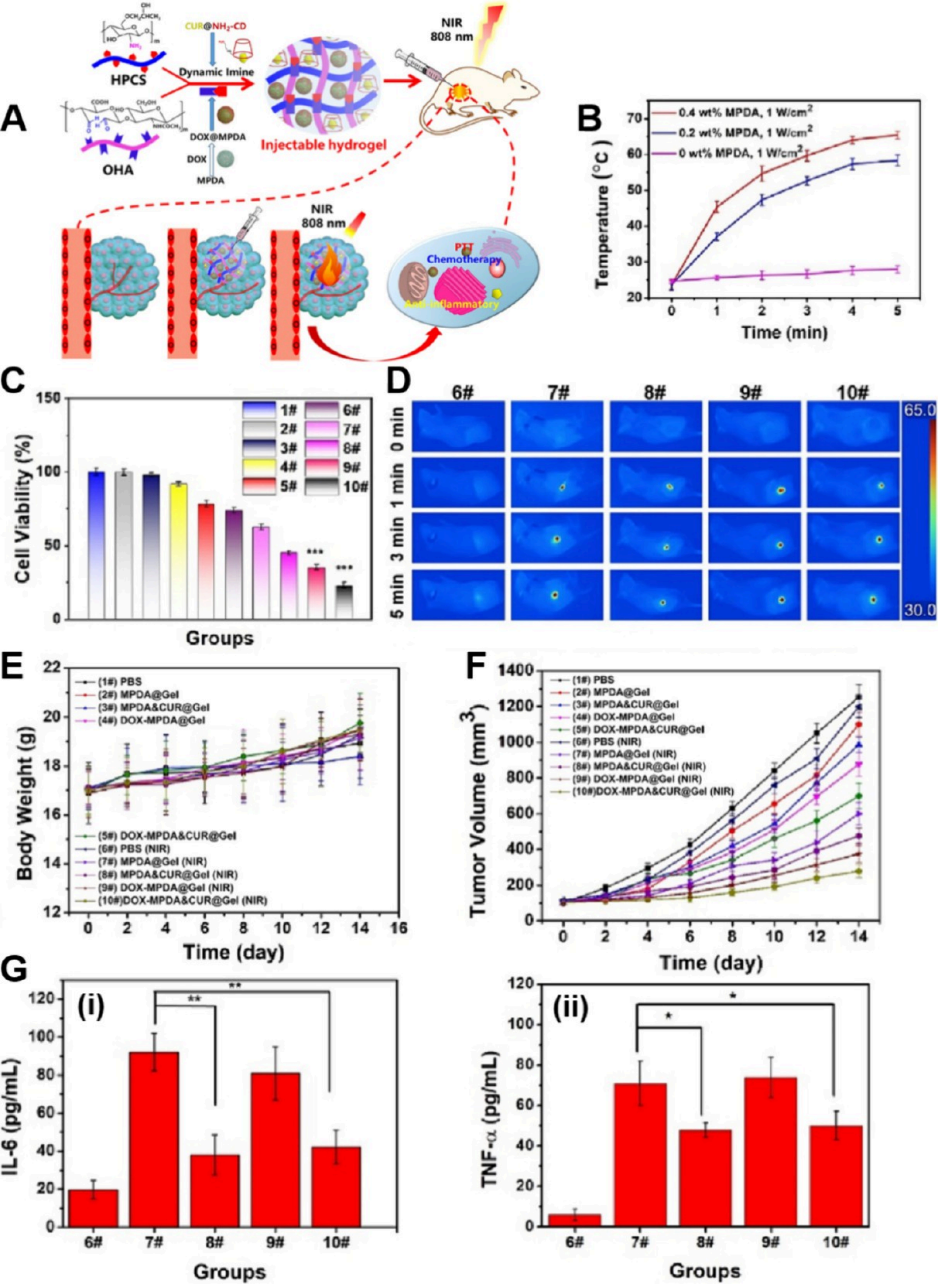
Preparation and antitumor capacity of MPDA composite hydrogels. (A) Schematic illustration for the design of the composite hydrogel for the tumor therapy with NIR irradiation. (B) The temperature change curve (ii) of the composite hydrogel with various composition of MPDA nanoparticles recorded with 808 nm NIR laser irradiation (1 W/cm^2^) for 5 min. (C) Cell viability with various composite hydrogels. 1#: Control, 2#: Gel, 3#: MPDA@Gel, 4#: CUR@Gel, 5#: DOX-MPDA@Gel, 6#: DOX-MPDA&CUR@Gel, 7#: MPDA@Gel + NIR (4 min), 8#: DOX-MPDA@Gel + NIR (4 min), 9#: DOX-MPDA&CUR@Gel + NIR (4 min), 10#: DOX-MPDA&CUR@Gel + NIR (6 min). (D) The images illustrated the temperature change of mice tumors injected with various samples under 808 nm NIR irradiation (1 W/cm^2^, 5 min). 6#: PBS (NIR), 7#: MPDA@Gel (NIR), 8#: MPDA&CUR@Gel (NIR), 9#: DOX-MPDA@Gel (NIR), 10#: DOX-MPDA&CUR@Gel (NIR). The change of body weight (E) and the tumor volume (F) of mice during the whole treatment (*n* = 5). G) IL-6 (i) and TNF-α (ii) levels with various treatments under the NIR irradiation in sera of mice. 6#: PBS (NIR), 7#: MPDA@Gel (NIR), 8#: MPDA&CUR@Gel (NIR), 9#: DOX-MPDA@Gel (NIR), 10#: DOX-MPDA&CUR@Gel (NIR). (*p ≤ 0.05, **p ≤ 0.01) [Reproduced with permission from ref 274. Copyright 2023, Elsevier Ltd.].

Wang et al.^275^ cross-linked 7-ethyl-10-hydroxycamptothecin (SN38)-loaded polydopamine (PDA) NPs in a poly­(ethylene glycol) (PEG) hydrogel. This PDA-SN38/PEG hydrogel exhibited minimal SN38 release under physiological conditions. However, upon irradiation with near-infrared light, the PDANPs showed excellent photothermal ablation ability, which enabled the release of SN38 from the gel in an ″off-on″ manner for tumor chemotherapy, effectively inhibiting tumor growth. Zhang et al.^276^ prepared a composite suspension by incorporating multiwalled carbon nanotubes and DOX with photothermal properties, which were then added dropwise to a poly-L-lactic acid (PLLA) solution. Subsequently, they fabricated DM fibers utilizing electrostatic spinning technology. These DM fibers exhibited slow and minimal DOX release under slightly acidic conditions at 37 °C due to the glass transition temperature of PLLA at 61.3 °C. However, upon exposure to infrared irradiation, the temperature rise triggered the sudden release of DOX, leading to excellent cokilling capabilities in both in vivo and in vitro models.

### 5.2 Photothermal-Immunotherapy

Certain tumors, such as advanced head and neck squamous cell carcinoma, are not sensitive to immunotherapy. Thus, relying solely on immunotherapy proves challenging in completely suppressing tumor growth. In contrast, PTT can trigger the immunogenic death of tumor cells, leading to the release of tumor-specific antigens. This, in turn, can activate the antitumor immune response and facilitate the penetration of immune cells, thereby improving the effect of immunotherapy.^277^ Although PTT can induce the body’s immune response, the depth of tumor and the difficulty of addressing distant tumor metastasis and recurrence limit its application. Thus, the combination of PTT and immunotherapy emerges as a promising therapeutic strategy for tumors.^278^

Zhou et al.^279^ loaded AuNCs containing aPD-1 antibodies into oxidized bacterial cellulose (OBC) hydrogels, which were modified using thrombin (TB). This resulted in the formation of TB/aPD-1@AuNCs/OBC hydrogel. The TB/aPD-1@AuNCs/OBC hydrogel exhibited excellent photothermal conversion efficiency when exposed to near-infrared irradiation, leading to thermal apoptosis in tumor cells. Additionally, it released aPD-1, thereby blocking the PD-1/PD-L1 pathway and promoting both local and systemic antitumor immune responses. This demonstrates its efficacy in photothermal ablation and significant inhibition of tumor recurrence.

However, immune checkpoint inhibitors alone often suffer from a low response rate. To address this issue, Jia et al.^280^ used resiquimod (R848), an agonist of TLR-7/8, and CPG ODNs, an agonist of TLR-9, which can activate dendritic cells (DCs) and induce the release of pro-inflammatory cytokines through multiple TLRs, and synthesized the RIC NPs together with indocyanine green (ICG) using a self-assembling technique. The resulting RIC NPs were then incorporated into an injectable PLEL hydrogel, yielding the RIC NPs@PLEL hydrogel (Figure 10 A).

**Figure 10:**
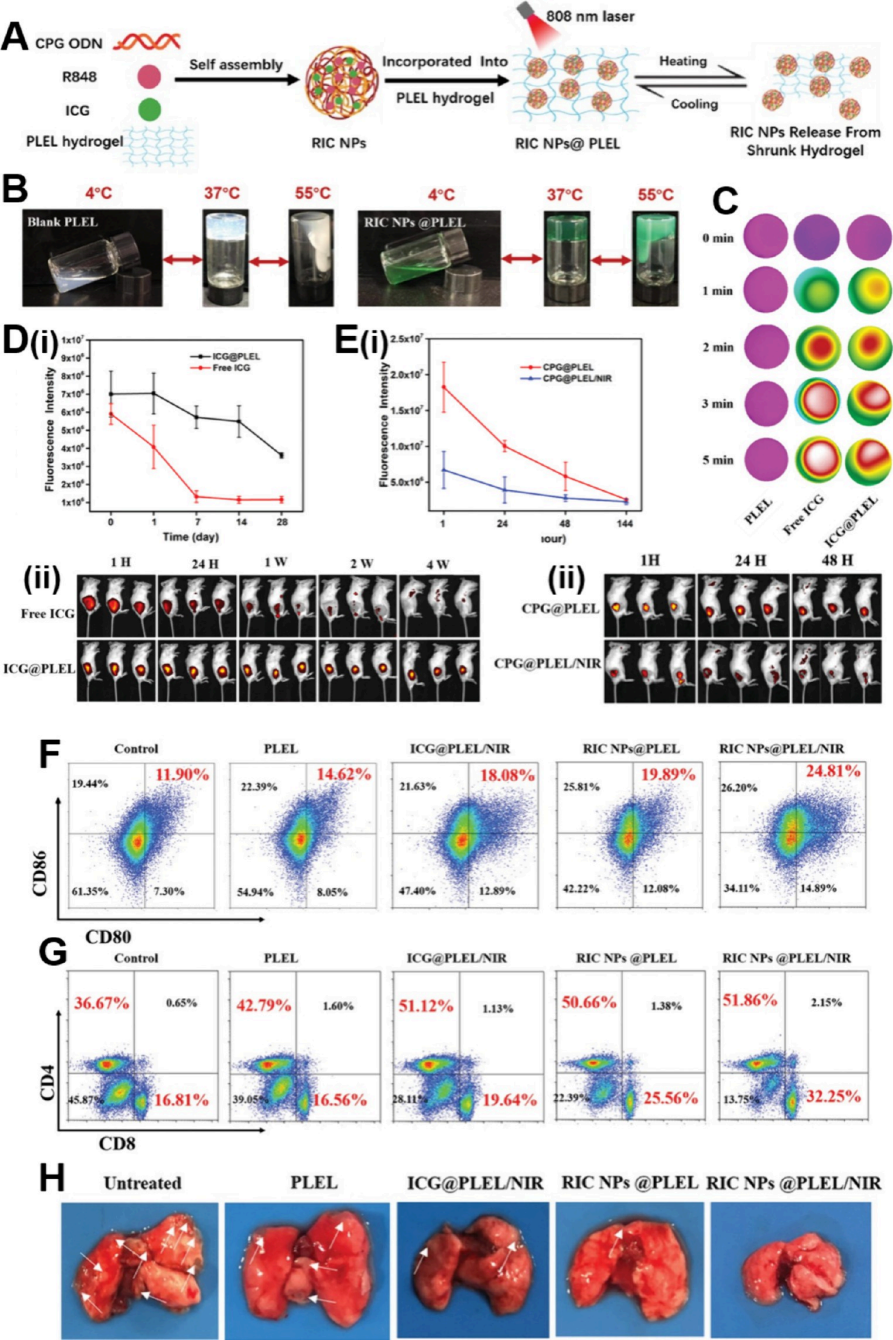
Preparation and antitumor capacity of RIC NPs@PLEL Hydrogels. (A) Schematic illustration of the preparation process of RIC NPs@PLEL hydrogels. (B) Reversible sol–gel transformation photos of PLEL hydrogels and RIC NPs@PLEL hydrogels. (C) Near infrared imaging of hydrogels under 808 nm laser irradiation. (D) In vivo extended release of ICG from ICG@PLEL hydrogel recorded by IVIS (ii) and quantitative analysis (i). (E) In vivo controlled release of CPG from PLEL hydrogel recorded by IVIS (ii) and quantitative analysis (i). Representative flow cytometry plots showing (F) matured DCs in lymph node and (G) cytotoxic CD3 + CD8 + T cells and CD3 + CD4 + T cells in spleens after various treatments. (H) Typical photographs of 4T1 metastatic foci for mice after different treatments. [Reproduced with permission from ref 280. Copyright 2020, WILEY-VCH Verlag GmbH & Co. KGaA, Weinheim]

It is noteworthy that the hydrogel undergoes a reversible phase transition. At temperatures ranging from 4 to 37 °C, the hydrogel remains in a liquid state, but when the temperature exceeds 37 °C, it gradually transforms into a gel. Moreover, within the temperature range of 45 to 55 °C, it gradually transitions into a sol state. This property allows the RIC NPs@PLEL gel, when locally injected, to shift from a liquid state (room temperature) to a gel state (approximately 37 °C). This transition is conducive to the creation of a localized drug reservoir (Figure 10 B). Upon exposure to near-infrared laser irradiation, ICG converts light energy into heat energy (Figure 10 C). The resultant high temperature promotes the further transformation of the gel into a soluble state (above 45 °C) while simultaneously photothermally ablating the tumor. This process triggers the on-demand release of R848 and CpG ODNs. When the ICG@PLEL hydrogel was subcutaneously injected into normal mice, fluorescence in vivo imaging system (IVIS) imaging showed that ICG maintained strong fluorescence intensity locally for an extended period of 4 weeks. In contrast, the fluorescence signals in the mice injected with the ICG solution rapidly diminished within 24 h, demonstrating the effective ability of the PLEL hydrogel to prolong the localized targeting of the drug (Figure 10 D, 10 E). Flow cytometry showed that the ICG@PLEL/NIR group slightly promoted DC maturation compared to the control group. This enhancement can be attributed to the NIR-induced release of tumor antigens. Conversely, the RIC NPs@PLEL/NIR group activated 24% DC maturation, resulting in a significant one-fold increase in the percentage of CD8+ T cells (Figure 10 F, 10 G). In addition, only 80% of mice in the RIC NPs@PLEL/NIR group achieved complete remission, whereas mice in all other groups showed varying degrees of recurrence (Figure 10 K). Thus, RIC NPs@PLEL hydrogel-mediated photothermal ablation, coupled with its triggered release of in situ vaccine-like functionality, consistently and effectively induced systemic immune responses. This approach successfully inhibited tumor metastasis and recurrence.

### 5.3 Photothermal-Chemodynamic Therapy

The efficacy of CDT, such as Fenton therapy, depends on the intensity of the chemical reaction involved. This intensity rises with increased temperature, whereas PTT accelerates the reaction process of CDT, leading to an increased production of ROS, thus enhancing the efficacy of CDT. Consequently, some researchers have designed synergistic therapies that combine PTT and CDT.

In contrast to the conventional Fenton reaction, Kim et al.^281^ used a Cu^2+^-mediated Fenton-like reaction for CDT. They incorporated Cu^2+^ and ICG into a catechol-functionalized hyaluronic acid (HC) hydrogel. Upon NIR irradiation, this HC/Cu/ICG hydrogel enhanced the Fenton-like reaction by elevating the temperature, simultaneously achieving photothermal tumor ablation. This approach facilitated a more rapid depletion of GSH in the TME and increased the production of −OH radicals, leading to a significant inhibition of tumor growth.

Chen et al.^282^ initially prepared CuO_2_ NPs, which were subsequently mixed with poly­(vinylpyrrolidone) (PVP) and employed in microneedle (MN) systems through a straightforward vacuum casting process in a mold, resulting in the MN@CuO_2_ system (Figure 11 A). For the MN system, the initial step involves skin penetration. It was demonstrated that the MN@CuO_2_ system possesses excellent strength up to 0.4 N/needle (Figure 11 B). This enabled easy penetration through the skin of pigs and mice, with complete release of the CuO_2_ loaded on the needle achieved within 1 min. The cells in the treatment group exposed to MN@CuO_2_ labeled with fluorescein isothiocyanate (FITC) showed obvious green fluorescence in vitro. This fluorescence intensity increased with the exposure time of CuO_2_ NPs (Figure 11 C). Furthermore, subsequent experiments demonstrated a continued increase in intracellular ROS levels following the administration of MN@CuO_2_ (Figure 11 D), while GSH levels exhibited a CuO_2_ quantity-dependent decrease (Figure 11 E), implying that the MN@CuO_2_ system can effectively perform Fenton-like therapies and deplete GSH for cell eradication. Experiments demonstrated that a system containing merely 10 wt % CuO_2_ could reduce cell activity to 10.5% (Figure 11 F). When irradiated with 1.0 W/cm^2^ NIR, the temperature increased by approximately 30 °C in 100 s. This temperature elevation exhibited a positive correlation with both CuO_2_ content and NIR power, reaching a maximum of approximately 60 °C. Moreover, the system maintained superior photothermal stability throughout five heating–cooling cycles (Figure 11 G). In vivo experiments demonstrated that the MN@CuO_2_ system could irradiate the melanoma area of mice with NIR at 0.75 W/cm^2^ for 10 min after piercing the skin in the melanoma-affected area. During this treatment, the maximum temperature reached 56.2 °C, with an exposure time exceeding 8 min above 50 °C. Although the MN@CuO_2_ group effectively inhibited tumor growth, the MN@CuO_2_/NIR group achieved even greater inhibition and in some cases even complete eradication of the tumor by simply increasing the duration of single irradiation session by 10 min (Figure 11 H, 11 I). Overall, this simple MN@CuO_2_ system successfully combines PTT and CDT using a single therapeutic agent. This approach has demonstrated significant antimelanoma effects while simultaneously mitigating potential drug-related toxic side effects. This finding provides a new reference for clinical melanoma treatment.

**Figure 11:**
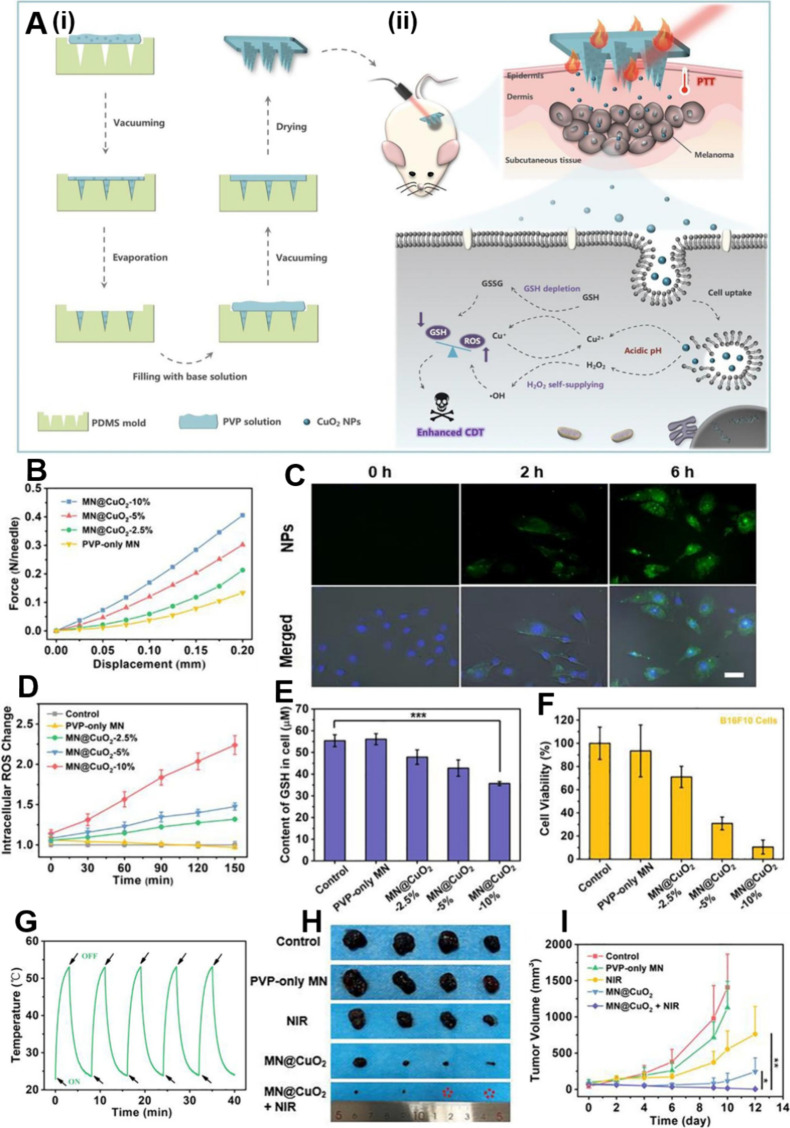
Preparation and antitumor capacity of MN@CuO_2_ system. (A) Schematic illustration of the preparation procedure and proposed therapeutic mechanism of MN@CuO_2_ system. (i) The preparation route of MN@CuO_2_ system based on a mold vacuum casting method. (ii) The antimelanoma effect mechanism of MN@CuO_2_ system by combining self-enhanced CDT with PTT. (B) Compressive strength tests of MN systems. (C) Time-dependent cell uptake of CuO_2_ NPs by B16F10 cells after treatment. Scale bar: 20 μm. (D) Intracellular ROS variations of B16F10 cells after different incubations. (E) Analysis of intracellular GSH of B16F10 cells after different incubations. (F) Cell viabilities of B16F10 melanoma cells incubated after different incubations for 24 h. (G) Temperature changes of MN@CuO_2_-10% system under the 808 nm laser irradiation (1.0 W·cm^–2^) for five heating–cooling cycles. (H) Photographs of the dissected tumors from different groups at the 10th or 12th day of therapies. (I) Tumor growth curves of the melanoma-bearing mice in different groups after treatments. (*p ≤ 0.05, **p ≤ 0.01, ***p ≤ 0.001) [Reproduced with permission from ref 282. Copyright 2021, Elsevier B.V.].

### 5.4 Photothermal-Photodynamic Therapy

PSs can transfer light energy to molecular oxygen and generate ROS for tumor suppressing, which is the main therapeutic mechanism of PDT, but the local hypoxic environment of tumors is not conducive to the generation of ROS. PTT can improve the intratumoral blood supply, and increase the oxygen content, and, simultaneously, raise the temperature to increase the response rate of PSs, thus obtaining a more effective PDT.^283,284^

As designed by Xing et al.,^285^ AuNPs were employed as the photothermite; however, it is important to note that AuNPs were not directly introduced; rather, they were obtained through a self-assembly and mineralization process using (AuCl_4_)^−^ ions (Figure 12 A). Additionally, the authors introduced a novel concept of a self-assembled injectable collagen-based hydrogel triggered by biomineralization. This process involves electrostatic complexation between positively charged collagen chains and inorganic anion clusters, such as (AuCl_4_)^−^ ions. Subsequently, the formed AuNPs serve as cross-linkers, influencing the mechanical properties of the gels through noncovalent interactions between the collagen chains and the AuNPs. Furthermore, during the synthesis of this collagen-based hydrogel, the size distribution of AuNPs in the hydrogel can be adjusted by varying the initial concentration of added HAuCl_4_ (Figure 12 B), thus affecting various properties, such as mechanical properties. Moreover, due to the noncovalent interactions between AuNPs and collagen chains, the hydrogel exhibits self-repairing capabilities, allowing it to undergo in situ repair and reorganization after intratumoral injection, ultimately forming a drug reservoir (Figure 12 C). Consequently, the hydrogel can provide sustained tumor therapeutic factors over an extended period. The photothermal performance was also excellent. The hydrogel containing 2 mM HAuCl_4_ reached 40 °C within 1.5 min under laser irradiation at approximately 0.17 W/cm^2^. Within 10 min of irradiation, it reached a maximum temperature of 55.5 °C (Figure 12 D). However, relying solely on PTT was insufficient. Therefore, MesoTetra (*N*-methyl-4-pyridyl) porphine tetrachloride (TMPyP) was introduced into the gel as a photodynamic therapeutic agent in vivo. All mice received intratumoral injections of collagen-based hydrogels containing TMPyP and AuNPs, but they underwent varying durations of irradiation (Figure 12 E). Tumor control was not effective in mice that received single session of irradiation, whereas tumor growth was significantly delayed in the other three groups that received multiple irradiations, with the group exposed to the highest number of irradiations even achieving complete tumor elimination (Figure 12 F, 12 G). Thus, the combination therapy of AuNPs with TMPyP following NIR irradiation demonstrated excellent PTT and PDT combination effects. and exhibited superior in vivo stability and sustained release, enabling multiple injections for repeated treatments, successful tumor growth inhibition, or even complete tumor eradication.

**Figure 12:**
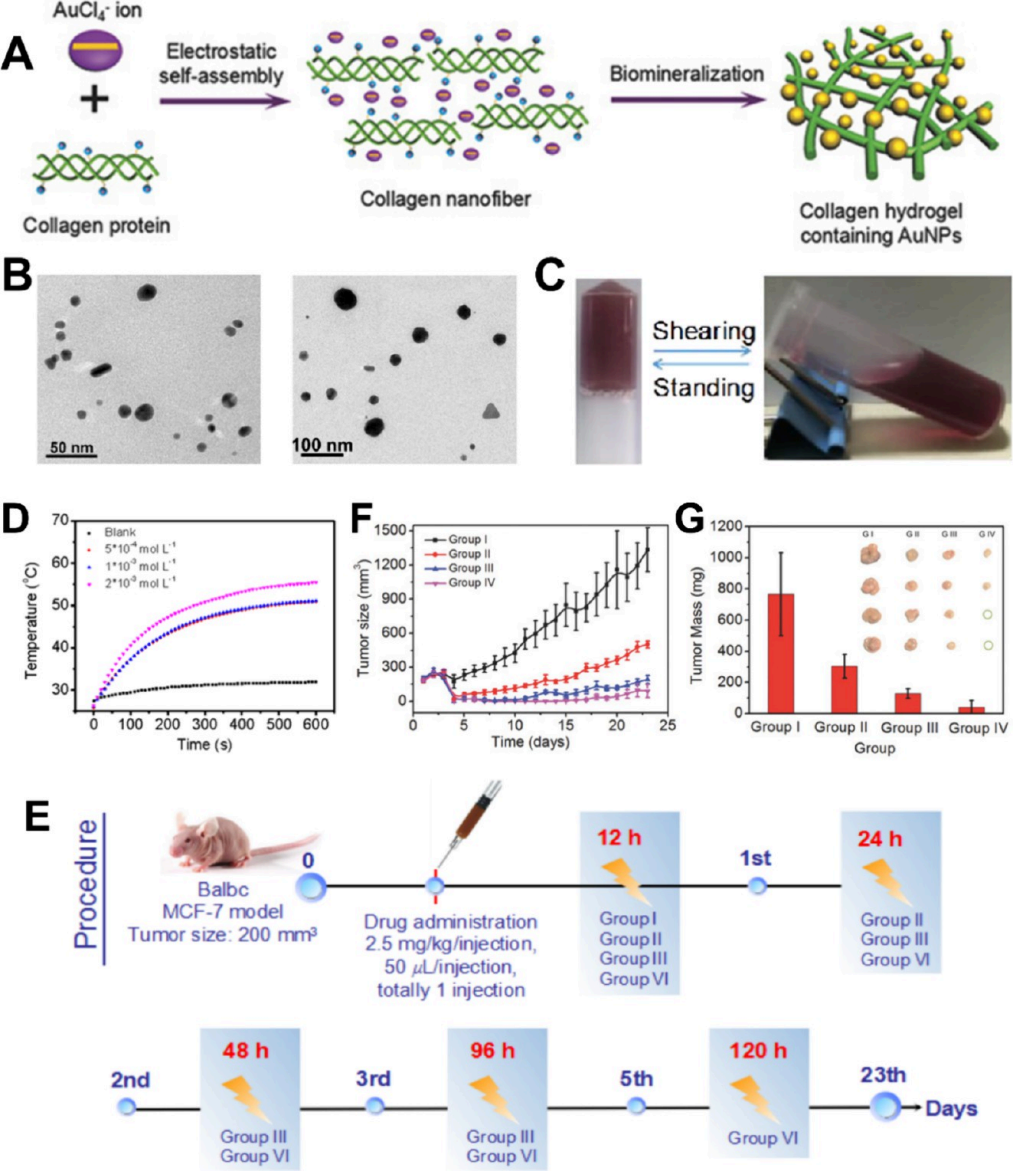
Preparation and antitumor capacity of collagen hydrogels containing AuNPs. (A) Schematic diagram of the fabrication of an injectable collagen-based hydrogel containing gold nanoparticles based on a biomineralization-triggered self-assembly process. (B) TEM images of AuNPs synthesized locally in the collagen hydrogels, which were prepared with the final chloroauric acid concentration 0.5 mM (Left) and 1 mM (Right), respectively. (C) Photograph of the gel showing shear thinning and self-healing properties. (D) The photothermal effect of the collagen hydrogels containing AuNPs after laser irradiation (635 nm, with a power intensity of 169.85 mW·cm^–2^ for 10 min) in vitro. Note: the collagen hydrogels were prepared with chloroauric acid concentration of 5 × 10^–4^, 1 × 10^–3^, 2 × 10^–3^ M, respectively. (E) Schematic diagram of the experimental procedure. (F) Tumor volumes of mice after different treatments. (G) Tumor weights of different treatment groups (inset: photographs of the resected tumors from each group obtained after 23 d of treatment). All data are presented as mean ± SD (*n* = 4). [Reproduced with permission from ref 285. Copyright 2020, WILEY-VCH Verlag GmbH & Co. KGaA, Weinheim].

### 5.5 Combination of Multiple Therapies

The objective of combining multiple therapies is to achieve synergistic tumor effects and to suppress tumors in multiple ways and from multiple perspectives, rather than simply superimposing therapeutic effects. This approach requires that the mechanisms of the employed therapies be related to each other. For example, PTT relies on exogenous NIR, limiting its impact to the photothermal zone and posing challenges in affecting deep-seated tumors. However, PTT can elevate the local tumor temperature and activate the body’s tumor immunity at the same time it performs photothermal ablation. This can be integrated with CDT or immunotherapy to compensate for PTT’s limitations, thus enhancing its therapeutic efficacy. Although chemotherapy can rapidly reduce initial tumor volume, it faces the challenges of tumor resistance and long-term toxic side effects. Therefore, supplementing chemotherapy with PTT, PDT, or immunotherapy not only efficiently addresses drug-resistant tumors through alternative approaches but also reduces the required dosage of chemotherapeutic drugs, mitigating toxic side effects. In conclusion, when employing multiple therapies to treat tumors, it is imperative to understand the advantages and shortcomings of each therapy, mitigating their limitations to achieve synergistic effects in tumor treatment.

For example, Zhou et al.^286^ developed a hydrogel combining PTT, CDT, and ST by cross-linking sodium ALG with Fe^3+^ and incorporating MoS_2_ nanosheets and glucose oxidase (GOx) to create the MoS_2_/ALG-Fe/GOx (MAF/GOx) hydrogel ( Figure 13 A).

**Figure 13:**
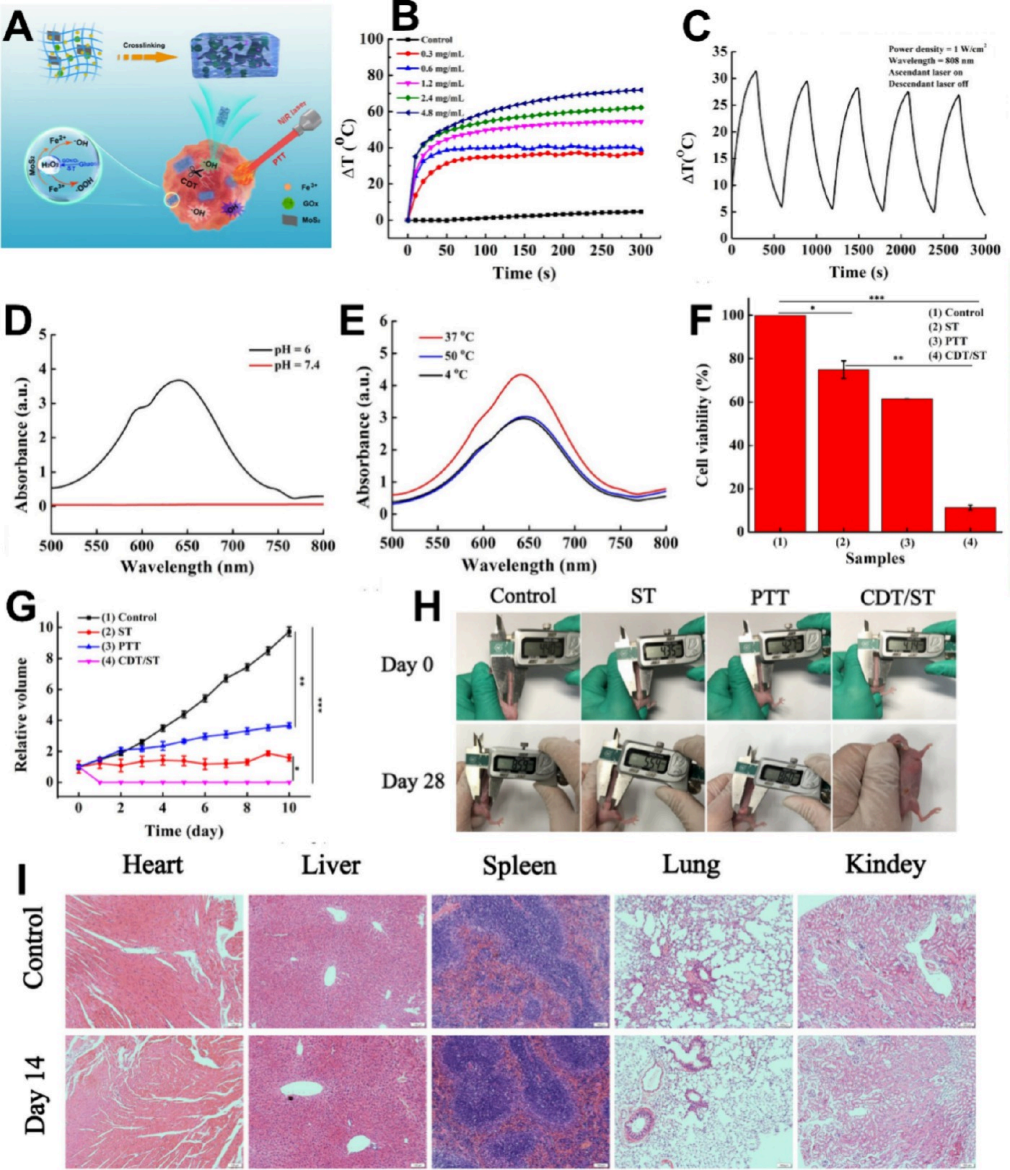
Preparation and antitumor capacity of MoS_2_-ALG-Fe/GOx Hydrogel. (A) Schematic illustration of the MoS_2_-ALG-Fe/GOx Hydrogel. (B) The temperature changes of MAF hydrogel with different MoS_2_ doping concentrations and the control group. (C) The photothermal stability assay of MAF hydrogel in saline. (D) UV–vis-NIR spectra of TMB chromogenic MAF/GOx hydrogel glucose solutions at the pH of 6.0 and 7.4. (E) UV–vis-NIR spectra of TMB chromogenic MAF/GOx hydrogel glucose solutions that were cultivated at different temperature. (F) HT29 cells viabilities that received different treatments. (G) Relative tumor volume growth curves of mice treated with different materials. (H) Digital images of tumor size of mice at day 0 and the 28th day, corresponding to (G). (I) Images of H&E stained major organs of healthy KM mice (control) and mice that were embedded with MAF hydrogel for 14 days. (*p ≤ 0.05, **p ≤ 0.01, ***p ≤ 0.001) [Reproduced with permission from ref 286. Copyright 2020, Elsevier B.V.]

Among them, MoS_2_ facilitates a redox reaction with Fe^3+^, reducing it to Fe^2+^, which leads to hydrogel degradation and induces the Fenton reaction to eradicate tumor cells. GOx reacts with tumor glucose to generate gluconic acid and H_2_O_2_, raising H_2_O_2_ levels in the TME and enhancing ST, further boosting the efficacy of CDT. Upon NIR irradiation, MoS_2_ converts light energy into thermal energy for PTT, increasing temperature and cell membrane permeability, which enhances the therapeutic reactions and overall efficacy. Simultaneously, the hydrogel matrix protects GOx from denaturation by the heat generated by MoS_2_.

The MAF/GOx hydrogel exhibits excellent photothermal performance, with a MoS_2_ nanosheet concentration of 0.3 mg/mL increasing the hydrogel’s ΔT by up to 36.9 °C at 1 W/cm^2^ NIR intensity ( Figure 13 B). It also shows superior photothermal stability over five NIR on/off cycles ( Figure 13 C). When immersed in glucose solution and subjected to temperature elevation or mildly acidic environment, the hydrogel promotes increased −OH production ( Figure 13 D, 13 E), suggesting that the photothermal or mildly acidic conditions of TME enhance the synergistic effects between ST and CDT.

In vitro, MAF/GOx reduces cell activity to approximately 10% even without NIR irradiation ( Figure 13 F), and in vivo, it prevents tumor recurrence during a 28-day treatment period, indicating effective tumor growth inhibition ( Figure 13 G to 13 I). While in vivo experiments combining MAF/GOx hydrogel with NIR were not conducted, it is possible that the hydrogel alone has potent tumor-killing capabilities, resulting in complete tumor inhibition without the need for additional NIR experiments. Overall, the MAF/GOx hydrogel demonstrates the potential to synergize PTT, CDT, and ST for effective tumor treatment, offering a novel strategy for efficient tumor therapy.

## 6 Summary and Prospects

In conclusion, LDDS, as a tumor-targeted therapeutic system, can release diverse therapeutic factors locally in tumors in a sustained, efficient, and cost-effective manner. However, their application and therapeutic mechanism still require further improvement. Treating them solely as drug delivery vehicles is a limited perspective, and LDDSs possess considerable untapped potential for future development. Here, we provide a concise overview of a few common LDDSs (Table 1).

**Table 1:** Information about Major LDDSs

systems	LDDS material	therapeutic agent	method of administration	in vitro/in vivo model	highlight	ref
fiber	PLLA	DOX	implant	human breast cancer cells (MDA-MB-231)/MDA-MB-231 xenograft BALB/c mice	drug release in two phases—burst release in the early stage and sustained release at a later stage	^287^
	PCL and poly(caprolactone-co-glycerol-monostearate)	N,N-dimethylformamide (DMF)	implant	Lewis lung carcinoma (LLC)/LLC allograft C57BL/6 mice	The fiber meshes exhibit superhydrophobicity, that sustains the release of cisplatin in a linear fashion over ∼ 90 days. The structure provides mechanical stability and flexibility in lung resection procedures.	^54^
hydrogel	alginate	Au/Ag nanorods (NRs) and DOX	inject	human nonsmall-cell lung cancer cells (A549)/A549 xenograft BALB/c nude mice	They explore an injectable hydrogel for combination PTT and chemotherapy, resulting in the complete eradication of tumors in most of the treated mice.	^288^
	peptide hydrogel	DOX	inject	MDA-MB-231 and 4T1/MDA-MB-231 xenograft BALB/c mice and 4T1 allograft BALB/c mice	They generate an peptide hydrogel, which self-assembles in the presence of chemotherapeutic drugs such as Dox to form well-organized structure.	^289^
scaffold	polymerized alginate	collagen-mimetic peptide GFOGER	implant	4T1, murine melanoma cell line (B16F10) and mouse ovarian epithelial cancer cells (ID8)/4T1 allograft BALB/c mice and ID8 allograft Albino B6 mice	They establish that appropriately designed polymer delivery systems can substantially improve the ability of anticancer immune cells to eradicate tumors.	^107^
	caffeic acid, nanoclay, alginate and chitosan	carbon dots-curcumin	implant	human breast cancer cells (MCF-7) and mouse embryonic fibroblasts (3 T3)/ MDA-MB-231 xenograft NSIG mice	This scaffold can effectively reduce the inflammatory reaction of postoperative wound, increase the production of neovascularization and collagen, inhibit postoperative tumor recurrence, and promote wound healing.	^290^
sponge	alginate, gelatin and PCL	curcumin	implant	MCF-7/murine ascites tumor cells (S180) allograft KM mice	This sponge stops bleeding quickly and prevents tumor recurrence after surgery through the controlled release of curcumin.	^291^
	chitosan, gelatin, poly(D,l-lactide) and PCL	DOX and triptolide	implant	n.a./H22 allograft BALB/c mice	This sponge can not only effectively prevent bleeding after tumor resection and exhibit antibacterial capability, but also releases dual drugs to significantly inhibit the tumor recurrence.	^292^
film	fibroin	DOX	implant	MDA-MB-231/MDA-MB-231 xenograft NOD/SCID mice	They investigate the release rate of DOX by changing β-sheet contents in the films, and demonstrate that the film provided better efficacy than systemic chemotherapy.	^56^
	poly(glycerol monostearate co-ε-caprolactone)	paclitaxel	implant	LLC, human large cell (NCI-H460), and Human mucoepidermoid (NCI-H292)/ LLC allograft C57BL/6 mice	This paclitaxel film significantly increased local drug concentration at the site of surgical resection and further reduced the rate of local tumor recurrence compared to systemic administration.	^135^
microneedle system	hyaluronic acid	aPD-1 and 1-methyl-dl-tryptophan (1-MT)	attach	n.a./murine melanoma cell line (B16F10) allograft C57BL/6 mice	transcutaneous immunotherapy using microneedle patch delivery of aPD-1 and 1-MT to increase local drug concentration while reducing side effects associated with immunotherapy	^293^
	polyvinylpyrrolidone and polyvinyl alcohol	cold atmospheric plasma (CAP) and aPD-1	attach	dendritic cells/B16F10 allograft C57BL/6 mice	They develop hollow-structured microneedle patch to facilitate CAP delivery through the skin and combine with aPD-1 for tumor therapy.	^294^
	Pluronic F127 and PEG	receptor 7/8 agonist (R848) and ovalbumin	attach	murine macrophage cells (RAW 264.7) and human colorectal cancer cells (HCT116)/mouse lymphoma cells (E.G7-OVA) allograft C57BL/6 mice	This microneedle patch utilizes the amphiphilic polymer Pluronic F127 to deliver R848 and ovalbumin and shows enhanced antigen-specific humoral and cellular immune responses as well as significant antitumor activity.	^295^

To begin with, the aforementioned underscores that fully exploiting the characteristics of LDDS-mediated fixed-domain tumor therapies can establish their prominent presence in the field of tumor therapy in the future, surpassing conventional drug delivery and SDDSs. In summary, LDDSs offer several significant advantages: 1) Drugs administered via LDDSs are released in situ within the tumor, significantly increasing the concentration of the drug in the tumor tissue. 2) LDDSs locally confine oncology drugs within the tumor, obviating the need for reliance on the circulatory system for biodistribution. This minimizes undesired drug accumulation in off-target tissues and reduces toxic side effects on adjacent healthy tissues, organs, and the entire body. 3) LDDSs are available in a wide range of material forms, facilitating the realization of desired properties and simpler preparation for achieving controlled and sustained drug release. 4) LDDSs enhance the bioavailability of drugs, allowing for improved therapeutic outcomes with fewer doses of drugs. 5) LDDSs sustain elevated local drug concentrations within the tumor for a long time, thereby reducing the local recurrence rate of tumor substantially. 6) LDDSs facilitate easier and more stable synergy among different therapeutic approaches.

Certainly, there are still limitations associated with LDDS-mediated tumor therapies in practical applications, especially when transitioning to clinical settings:^296–299^ 1) ^1^O_2_ has a lifetime of approximately 0–3 μs in aqueous solutions and a limited diffusion range of 0–20 nm. Consequently, therapies primarily reliant on ROS for tumor eradication may not be able to achieve effective treatments. 2) Therapies employing CDT and PDT to activate endogenous substances for tumor elimination may fail to yield the anticipated therapeutic effect, primarily because of the low oxygen levels and weak acid characteristics of the TME, which make it challenging to sustain continuous CDT and PDT treatment. The therapeutic effects of these therapies diminish markedly when endogenous ROS or H^+^ are depleted. 3) Owing to the weak penetration ability of NIR, light-dependent therapies, such as PTT and PDT, face difficulties in delivering efficient tumor treatment or controlled drug release for deep-seated tumors. In addition, LDDSs are not perfect per se, and their specific administration modalities can limit their applicability:^124,125,300–304^ 1) LDDSs form local reservoirs when administered intratumorally or peritumorally, which confers macroscopic targeting of the therapies but restricts therapeutic agents to the tumor site, rendering them ineffective against distant metastases or subtle lesions. 2) Challenges associated with anatomical structure or the inability to precisely localize the lesion, making it difficult to directly deliver the drug locally. 3) When LDDSs are injected into the tumor or implanted via minimally invasive surgery, they may inflict some damage to the tumor compared with intravenous administration in practice and are more likely to lead to tumor cell dissemination into the bloodstream, elevating CTC levels and the risk of both local recurrence and distant metastasis of the tumor. 4) Currently, aseptic principles are not consistently followed in the preparation of most of the LDDSs in the experimental stage. This noncompliance heightens the risk of infection during the implantation of these LDDSs into the body.

Even though LDDSs have considerable advantages, there are still only a few cases of successful clinical translation of LDDSs owing to their shortcomings and limitations. Therefore, the primary direction of future research should revolve around optimizing LDDSs, integrating them with research trends, and harnessing their characteristics to facilitate research breakthroughs and clinical applications. In the preceding sections, we summarized the shortcomings of LDDSs, and in the subsequent sections we outline a few possible solutions to these issues, thereby providing valuable insights for guiding future research.

To address the issues of short ROS lifespan and limited diffusion distance, it is proposed that a mitochondria-centric approach be adopted. Given the heightened sensitivity of mitochondria, the energy factories of the cell, to ROS damage compared with the cell membrane or cytoplasm, combining LDDSs with mitochondria-targeting technology for ROS generation in close proximity to or within the mitochondria could potentially resolve this concern. In addition, the challenge of insufficient endogenous substances in tumors may be mitigated through the development of specialized LDDSs, such as those with H^+^ or ROS as breakdown products or those capable of inhibiting the efflux of certain substances, such as lactic acid. This aims to improve the efficacy of CDT or PDT. Furthermore, while endoscopic fiber-optic diffusers and wireless implantable light systems address the issue of inadequate tissue penetration by NIR light, they also increase the risk of infection and immune rejection, operational complexity, and treatment costs.^305,306^ Increasing irradiation intensity to enhance penetration depth raises the risk of peritumor adverse effects.^307,308^ Therefore, in the future, specialized vibration waves, such as penetrating X-rays or ultrasound waves, may be used in conjunction with appropriate LDDSs and responsive devices to achieve deep PDT or SDT. Another avenue is the localized delivery of radionuclides to tumors using LDDSs, potentially inducing PDT via Cherenkov radiation (CR) without relying on an external light source. Nevertheless, precise control of the dosage of the X-rays and the CR poses a challenge.

As previously noted, LDDSs possess certain limitations. While addressing the issues of elevated risk for CTC and infection through enhancements in delivery methods, production processes, and the integration of diffusion molecular retention technology for oncology treatment is feasible, challenges persist when confronting systemic or imprecisely localized lesions. This remains an inevitable issue in LDDS’s application. In the subsequent discussion, we will provide some insights into enhancing their future applications.

Although LDDSs today offer various specialized features, such as responsiveness, adhesion, and blood- absorbing properties, and can be combined with diverse therapeutic agents for oncological treatment, they still face challenges in assuming a systemic therapeutic role, even when dealing with certain tumors such as Hodgkin’s lymphoma.^309–311^ Compared with localized treatments, chemotherapy or immunotherapy demonstrate superior control over distant metastases and subtle lesions. However, LDDS-mediated tumor therapy can deliver precise local treatment at a markedly reduced cost compared to surgery while maintaining the capability to completely eradicate local tumors. Nevertheless, it remains challenging to provide systemic treatment through LDDSs. Therefore, employing LDDSs as an alternative to surgical interventions, in conjunction with systemic therapy, may be a potential future direction in the development of LDDSs.

Tumor treatment with LDDSs could both complement and potentially replace surgical interventions. In the future, it may be possible to employ specialized LDDS post-tumor resection. These LDDSs could securely adhere to the incision margin, conform to its shape to staunch bleeding, absorb exudate without concern for bacterial contamination, and continuously release therapeutic agents in the surrounding area to prevent recurrence and metastasis. Such LDDSs would necessitate adhesion, deformability, support, absorbency, hemostatic capacity, and drug-loading capacity. Consequently, the development of multifunctional LDDSs, which combine these various properties, may represent a key future application scenario.

Furthermore, the currently used responsive LDDSs still require improvement. Externally controlled systems, such as light-responsive and ultrasound-responsive LDDSs, can achieve controlled drug release, but the drug release caused by slow degradation in vivo remains inevitable. On the other hand, LDDSs responsive to endogenous conditions resemble ″sustained-release capsules″ that load therapeutic agents. For example, pH-responsive LDDSs release the drug in response to the acidic tumor microenvironment. However, individual variations in the microenvironment and uncertainties in microenvironment changes post-treatment pose significant challenges in accurately calculating the endogenous H^+^ concentrations. These challenges make it difficult for current responsive LDDSs to precisely control drug release. Similarly, while LDDSs are generally synthesized from biocompatible materials, which may only cause mild inflammation or immune rejection, few studies have reported the impact of their metabolic and degradation products on tumor suppression. Ideally, the metabolites of LDDSs should aid tumor therapies or contribute to the recovery of the organism. For example, pH-responsive LDDSs synthesized from materials with lactate as a metabolic product could help when endogenous H^+^ is insufficient, or materials whose metabolic products assist bone healing could be used to synthesize LDDSs for treating bone tumors.

We have entered the era of molecular targeting in tumor therapy, with ongoing iterations of various targeted drugs. Compared with targeted therapy, LDDSs are similar to a macro-targeted drug delivery approach that offers reduced toxic side effects and economic burdens. Therefore, LDDSs could serve as a viable option for patients with tumors facing challenges such as a lack of effective targeted drugs, resistance to existing treatments, or financial constraints.

## ABBREVIATIONS

3D: three-dimensional
1-MT: 1-methyl-dl-tryptophan
AgNPs: silver NPs
AGS: alginate/gelatin sponges
ALG: alginate
AMF: alternating magnetic field
aPD-1: antiprogrammed death 1
BSA: bovine serum albumin
CAP: cold atmospheric plasma
CDT: chemodynamic therapy
CF: curcumin-loaded electrospun fibers
CFSC: cisplatin-loaded fiber/sponge composite
CH-DOX: chitosan-loaded DOX
CO: carbon monoxide
CPMV: cowpea mosaic virus
CR: Cherenkov radiation
CTC: circulating tumor cells
CUR: curcumin
DCs: dendritic cells
DDSs: drug delivery systems
DMF: N,N-dimethylformamide
DOX-loaded: doxorubicin-loaded
FDA: Food and Drug Administration
FeMSN: Fe^0^ nanocrystal embedded MSN
FITC: fluorescein isothiocyanate
GAT: gas therapy
GET: gene therapy
GNPs: gold NPs
GOx: glucose oxidase
H2S: hydrogen sulfide
HC: hyaluronic acid
HPCS: hydroxypropyl chitosan
ICG: indocyanine green
IPN: interpenetrating polymer network
IVIS: in vivo imaging system
LDDSs: local drug delivery systems
LLC: Lewis lung carcinoma
MAF/GOx: MoS2/ALG-Fe/GOx
MDR: multidrug resistance
MN: microneedle
MPDA: mesoporous polydopamine
MSN: mesoporous silica NPs
MT: metabolic therapy
NIR: near-infrared
NO: nitric oxide
NPs: nanoparticles
NRs: nanorods
OBC: oxidized bacterial cellulose
OHA: oxidized hyaluronic acid
OH: hydroxyl radicals
PCL: poly­(epsilon-caprolactone)
PDA: polydopamine
PDT: photodynamic therapy
PEG: poly­(ethylene glycol)
PEG-DOX-Cur: poly­(ethylene glycol)-DOX-curcumin
PEO: poly­(ethylene oxide)
PG fibers: PCL-gelatin fibers
PLA: poly­(l-lactide)
PLGA: poly­(d,l-lactide-co-glycolide)
PLGA–PTX: polylactide-co-glycolide PTX microspheres
PLLA: poly-l-lactic acid
PPa: pyropheophorbide
PS: photosensitizer
PTX: paclitaxel
PVP: poly­(vinylpyrrolidone)
R848: resiquimod
ROS: reactive oxygen species
SDDS: systemic DDS
SDT: sonodynamic therapy
siRNA: small interfering RNA
SN38: 7-ethyl-10-hydroxycamptothecin
ST: starvation therapy
TB: thrombin
VLPs: virus-like particles.
